# The chemoprotective hormetic effects of rosmarinic acid

**DOI:** 10.1515/med-2024-1065

**Published:** 2024-10-21

**Authors:** Edward J. Calabrese, Peter Pressman, A. Wallace Hayes, Gaurav Dhawan, Rachna Kapoor, Evgenios Agathokleous, Linda A. Baldwin, Vittorio Calabrese

**Affiliations:** School of Public Health and Health Sciences, Department of Environmental Health Sciences, Morrill I-N344, University of Massachusetts, Amherst, MA, 01003, United States of America; University of Maine, Orono, ME, 04469, United States of America; Center for Environmental Occupational Risk Analysis and Management, College of Public Health, University of South Florida, Tampa, FL, United States of America; Sri Guru Ram Das (SGRD), University of Health Sciences, Amritsar, India; Saint Francis Hospital and Medical Center, Hartford, CT, United States of America; School of Ecology and Applied Meteorology, Nanjing University of Information Science & Technology, Nanjing, 210044, China; 5 Sapphire Lane, Greenfield, MA, 01301, United States of America; Department of Biomedical and Biotechnological Sciences, School of Medicine University of Catania, Catania, 95123, Italy

**Keywords:** hormesis, dose–response, U-shaped dose–response, biphasic dose response, neuroprotection

## Abstract

Rosmarinic acid is a polyphenol found in numerous fruits and vegetables, consumed in supplement form, and tested in numerous clinical trials for therapeutic applications due to its putative chemopreventive properties. Rosmarinic acid has been extensively studied at the cellular, whole animal, and molecular mechanism levels, presenting a complex array of multi-system biological effects. Rosmarinic acid-induced hormetic dose responses are widespread, occurring in numerous biological models and cell types for a broad range of endpoints. Consequently, this article provides the first assessment of rosmarinic acid-induced hormetic concentration/dose responses, their quantitative features, mechanistic foundations, extrapolative strengths/limitations, and their biomedical, clinical, and public health implications.

## 1 Introduction

Rosmarinic acid is a phenolic compound found in species of Boraginaceae and Lamiaceae families, as well as in the leaves of rosemary (*Rosmarinus officinalis* L). It is also found in peppermint, lemon balm, oregano, sage, and thyme [1]. Rosmarinic acid is well known for its capacity to induce antioxidant, anti-inflammatory, pro-apoptotic, neuroprotective, and antitumor effects [2,3]. Due to its antioxidant effects, rosmarinic acid has become widely used as a dietary supplement. This article evaluates whether rosmarinic acid may act as a hormetic agent, mediating its chemoprotective effects as has been shown for similar agents, such as caffeic acid, a derivative of rosmarinic acid [4,5,6,7]. Initial attempts to identify articles displaying rosmarinic acid-induced hormetic effects via key word searches (e.g., hormesis/hormetic) in the Web of Science or Pub Med yielded extremely few entries. This created the need for alternative search strategies in order to obtain relevant papers. Since many hormetic papers were subsequently obtained via the use of complex alternative search strategies for rosmarinic acid, and since these obtained papers rarely used the terms hormesis or hormetic, it appears that these investigators were not familiar with the hormesis concept. Consequently, this paper includes a brief overview of the hormesis concept to introduce it to the rosmarinic acid research community.

## 2 Hormesis overview

**Figure 1:**
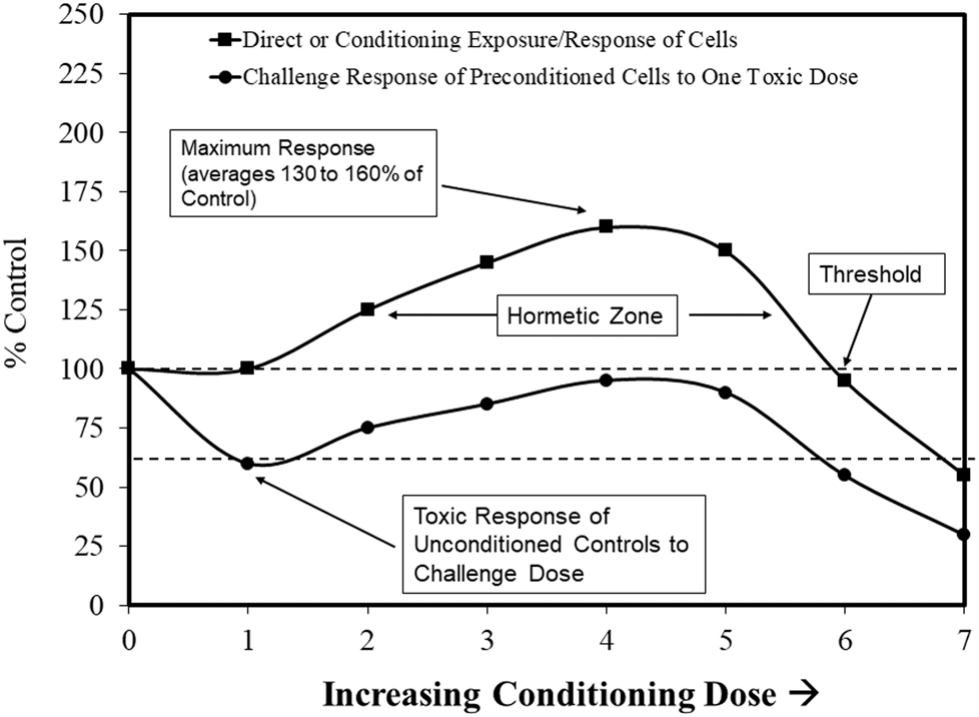
Dynamic features of the hormetic dose response.

Hormesis is an evolutionary-based biphasic dose/concentration response that often indicates an adaptation to low levels of endogenous and exogenous stress. It was initially reported in the biomedical literature in the late 1880s by Hugo Schulz [8,9]. The research of Schulz experimentally evaluated the effects of a wide spectrum of toxic agents on yeast metabolism. The hormetic responses show a low concentration/dose stimulation and a high concentration/dose inhibition (Figure 1) [8,9,10,11,12,13,14,15]. This concentration/dose–response pattern characteristic reveals specific quantitative features with a maximum stimulatory response, frequently 30–60% greater than unexposed control groups (100%). The hormetic stimulatory concentration/dose range is often 10–20-fold immediately below the usual toxicological or pharmacological thresholds. Nonetheless, the hormetic concentration/dose–response range frequently has substantial variability, often more than 50-fold and in a low proportion of cases (1–3%) exceeding 1,000-fold. The hormetic response can be induced within three types of experimental protocols: (1) a direct agent exposure; (2) a hormetic conditioning dose [given either prior to a toxic dose (preconditioning), concurrently with a toxic dose, or following a toxic dose (post-conditioning)] [16,17,18]; or (3) a modest overcompensation stimulation following what is typically an initial modest toxic response and/or a disruption in homeostasis [11,16]. Of biological importance is that the hormetic concentration/dose response has considerable generality [19,20,21,22,23], being independent of the biological model (e.g., microbes, plants, animal models, and humans), endpoint, level of biological organization (i.e., cell, organ, organism), *in vitro* and *in vivo* evaluations, inducing agent [9,11], and mechanism [24,25]. Hormesis offers an experimentally based framework to assess chemical mixtures, including additivity and synergism [11]. Numerous adaptive and/or beneficial hormetic effects that have been associated with low-dose exposures to various chemical and physical agents include lifespan extension, enhanced development and growth, diminished tumor incidence, improved resistance to infection, and tolerance to toxic substances and radiation [9]. The public health/environmental toxicology implications are as significant as those reported in the pharmacologic literature.

The hormetic dose–response concept therefore is a highly conserved resource-management, evolutionary-based, dose-response strategy that affects all species and cell types for numerous endpoints and describes both the quantitative limits of plasticity and the magnitude of stimulatory responses in constitutive/growth (anabolic) and adaptive (catabolic) processes [26]. The hormetic response, thus, is a fundamental component of anabolic and catabolic metabolism.

## 3 Memory

**Figure 2:**
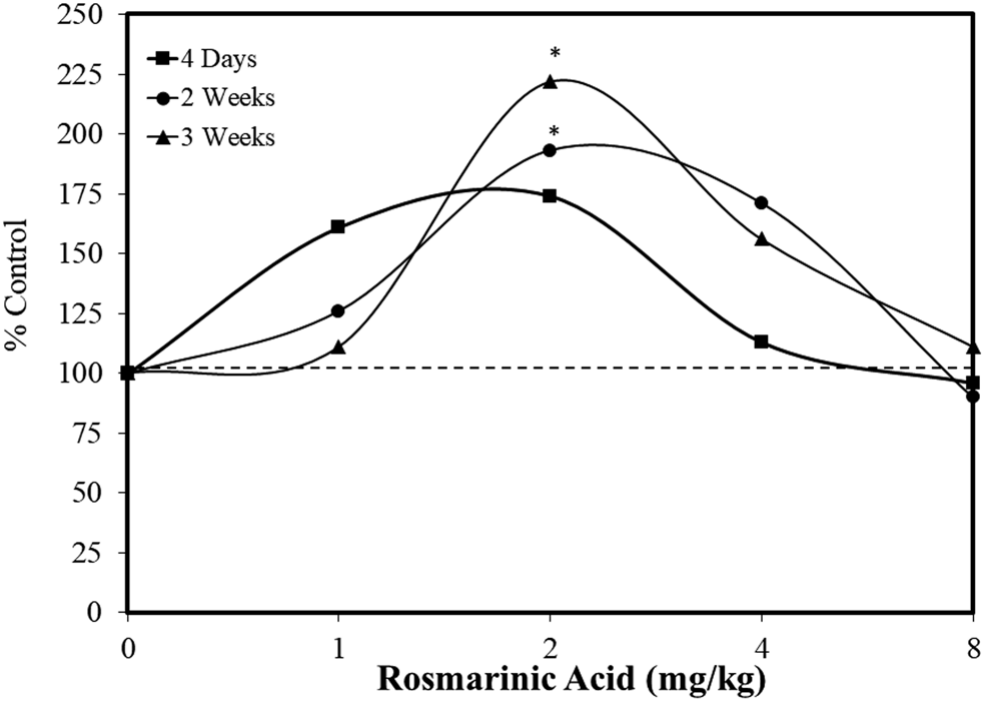
Rosmarinic acid enhanced memory in ICR male mice in the Morris water maize (escape latency) (modified from Park et al. [27]) (*= statistical significance *P* = <0.05).

In the case of cognitive performance, Park et al. [27] reported on the effect of rosmarinic acid on prolyl oligopeptidase (POP) activity. POP is a serine protease that cleaves a number of neuropeptides that have 30 or less amino acid residues at the carbonyl side of the internal proline residue. POP therefore can affect multiple critical molecules such as arginine vasopressin (AVP), substance P (SP), oxytocin, and angiotensin IV. While these agents have uniquely diverse biological profiles, they share a capacity to affect cognitive function in a number of ways (e.g., memory consolidation, storage, and retrieval, among others). Park et al. [27] hypothesized that increasing brain concentrations of AVP, SP, and angiotensin IV via POP inhibition may lead to an improvement in cognitive function. This research question then led to screening a large number of agents for POP inhibition. Rosmarinic acid effectively inhibited POP activity, thereby leading to the suggestion that it may augment cognitive functions as well as reduce the likelihood of some psychiatric disorders. Based upon this theoretical framework, Park et al. [27] assessed the effects of rosmarinic acid on cognitive performance in young adult male institute of cancer research (ICR) mice using the Morris water maze to assess hippocampal-dependent spatial memory. The intention was to assess whether rosmarinic acid (pretreatment 60 min prior to being tested) could enhance the capacity of the mice to remember and/or locate a submerged platform in order to escape the water pool into which they had been placed. Following an elaborate training and evaluative protocol, rosmarinic acid treatment (1, 2, 4, or 8 mg/kg – oral) was assessed for different periods (an acute, limited exposure duration of 4 days or subchronic, longer exposure durations of two or three weeks) within the water maze protocol. The rosmarinic acid treatment protocol enhanced the gradient crossings compared to control animals, showing an inverted U-shaped dose–response, with the optimal dosage being 2 mg/kg for each duration period (Figure 2) [27]. Since the number of crossings can be employed to assess memory performance, the experiment suggested that rosmarinic acid was active within this mouse model.

Rosmarinic acid, which was the first agent screened for suppression of POP activity for cognitive performance, enhanced this response during the acute and subchronic duration groups. The quantitative features of the dose responses suggested that the effect-induced magnitude and width were modestly larger in the subchronic duration studies. The authors acknowledged the inverted U-shaped dose-response, suggesting that higher doses of cholinomimetics may activate presynaptic autoreceptors, leading to the inverted U-shaped dose-response [28,29]. Since several endogenous peptides display the “paradoxical” decline of the cognition-enhancing effect at high concentrations [29], Park et al. [27] hypothesized that rosmarinic acid might induce the inverted U-shaped dose–response by increasing the concentration of neuropeptides such as AVP, SP, and angiotensin IV by its inhibition of POP.

Finally, it has been debated whether rosmarinic acid mediates its cognitive effects via a direct impact on the brain since it is generally recognized that it has a very low capacity to cross the blood–brain barrier (BBB) or via an alternative indirect mechanism. This issue will be addressed within the discussion section as it applies to other neural endpoints.

## 4 Anxiety

**Figure 3:**
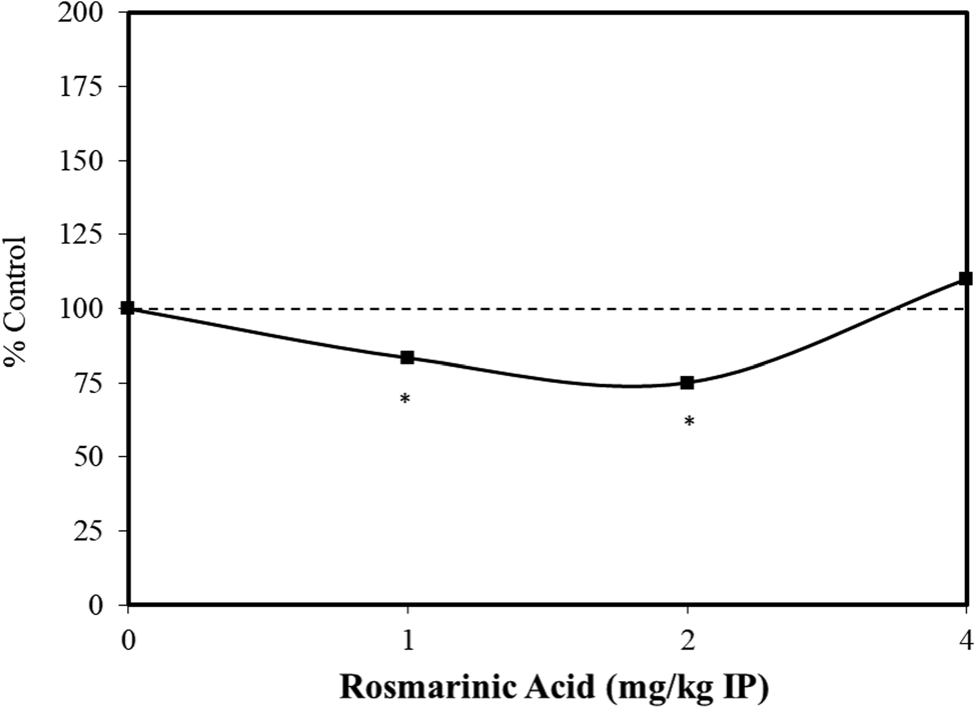
Effects of rosmarinic acid on forced swimming induced immobilization in ICR male mice (modified from Takeda et al. [31]) (*= statistical significance *P* = <0.05).

**Figure 4:**
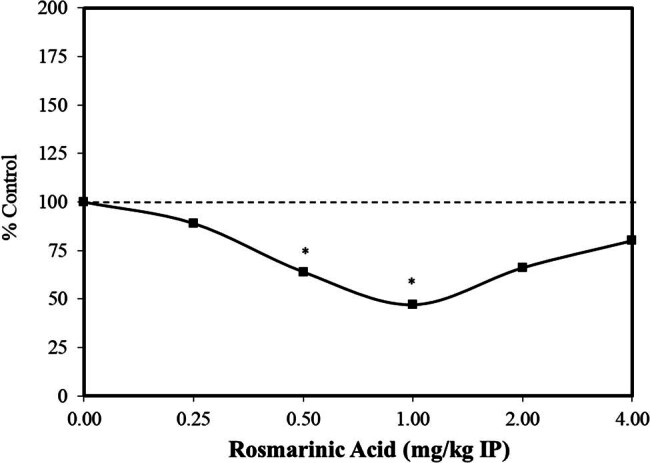
Effects of rosmarinic acid on the freezing behavior of male ddY mice exposed to conditioned fear stress (modified from Takeda et al. [32]) (*= statistical significance *P* = <0.05).

Extensive preclinical research has explored the nature of the dose–response for anxiety-related disorders. It has been common for such studies looking at anxiolytic-related effects to use laboratory protocols, such as the elevated plus maze test, the light-dark test, the tail suspension test, and the forced swimming test, to assess drug effects on anti-depression behaviors. In an assessment of dose–response features of possible effective agents using these types of experimental procedures, hormetic dose–response relationships were prominently reported [30]. While this analysis was principally directed toward pharmacological agents, various traditional oriental herbal medicine practices have been employed for the treatment of anxiety-related disorders. Of relevance to the present assessment is that leaf extracts of *Perilla frutescens*, containing rosmarinic acid, have been extensively used to treat anxiety-related disorders. These observations led to the assessment of rosmarinic acid to reduce anxiety-related behaviors with a focus on anti-depression-like effects. Using the forced swimming test [31] and a freezing behavior response based on intermittent inescapable electric foot shocks [32], rosmarinic acid treatments induced hormetic biphasic dose-response relationships, with optimal doses being similar for each endpoint (Figures 3 and 4) [31,32]. While the authors acknowledged that the mechanistic basis for the responses of rosmarinic acid was not known, the rosmarinic acid did not affect the uptake of monoamines by synaptosomes or the mitochondrial monoamine oxidase activity in the mouse brain. The authors raised the question of whether the observed effects may be related to antioxidant activities, including interactions with nitric oxide, affecting its production and release.

**Figure 5:**
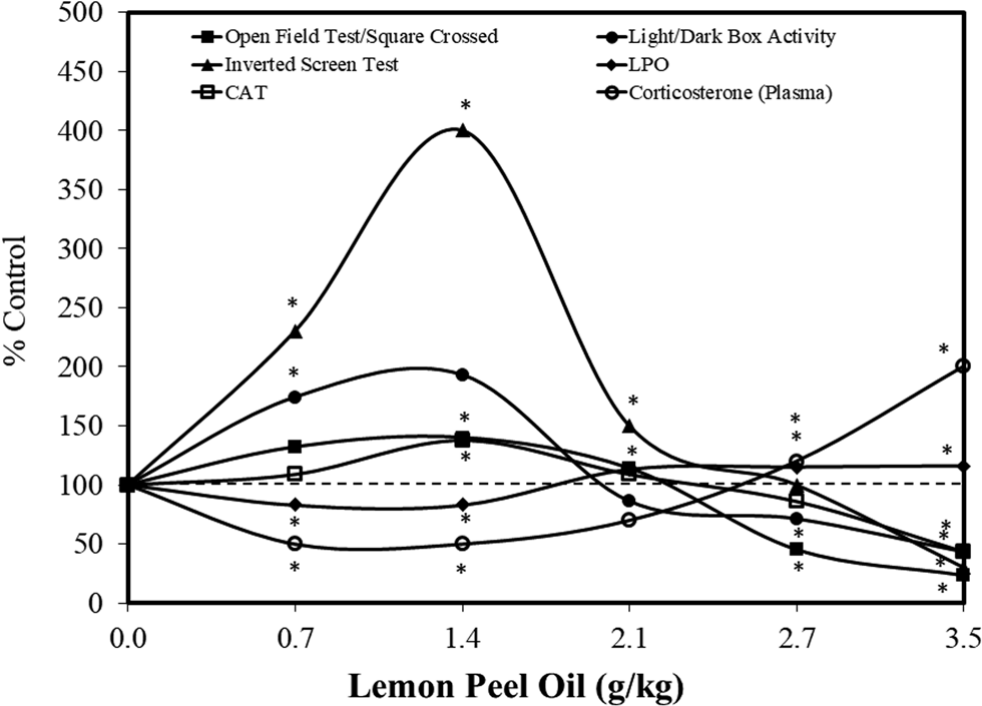
Effects of lemon peel oil on anxiety in SD rats (modified from Yousuf et al. [33]) (*= statistical significance *P* = <0.05).

While the above-discussed papers evaluated the effects of rosmarinic acid, Yousuf et al. [33] assessed the effects of lemon peel oil, which contains rosmarinic acid along with a mixture of other polyphenols, using a Sprague-Dawley (SD) rat model over a 14-day period (0.7, 1.4, 2.1, 2.7, 3.5 g/kg/day – oral). The lemon peel oil mixture induced a consistent series of hormetic dose responses for a series of standard behavioral tests and various measures of stress and antioxidant activities (Figure 5). The lower doses tested had a positive effect on the spectrum of anxiety-related behaviors. The authors suggested that the optimal effects reflected a balance between antioxidant and prooxidant effects.

## 5 Pain

**Figure 6:**
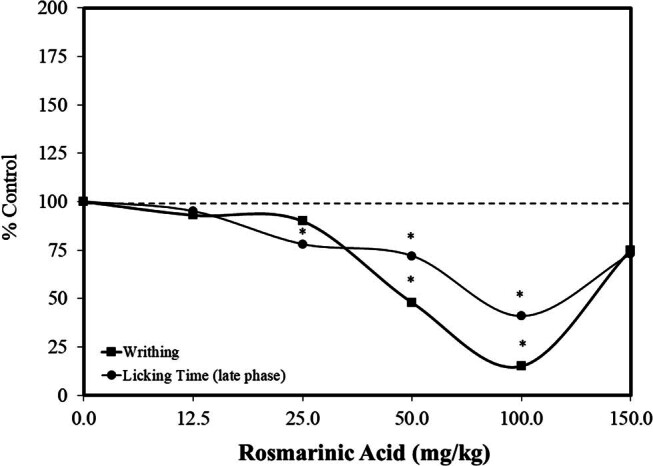
Effects of rosmarinic acid on pain response in male ICR mice (modified from Boonyarikpunchai et al. [36]) (*= statistical significance *P* = <0.05).

In 2008, Calabrese [34] assessed the occurrence of pain and U-shaped dose responses within a hormetic dose-response framework. This evaluation focused on three areas: the occurrence, mechanisms, and clinical applications of such hormetic dose responses. The assessment focused on traditional pharmacological agents, such as yohimbine, apomorphine, promethazine, L-dopa, prostaglandins, dopamine, cannabinoids, and opiates. However, traditional Asian medicine has commonly employed plant-based extracts in the treatment of pain, headaches, and related clinical phenomena [35]. Within this context, Boonyarikpunchai et al. [36] reported that rosmarinic acid affected the occurrence of pain in a mouse model using various standard pain-related experimental procedures (i.e., the hot plate, acetic acid-induced pain-related writhing, formalin tests as well as carrageenan-induced paw edema and cotton pellet-induced granuloma formation), consistently showing the occurrence of hormetic dose-response relationships (i.e., optimized at 50 and 100 mg/kg, administered orally 1 h prior to pain treatment) across the spectrum of endpoints studied (Figure 6). However, the doses used far exceeded those employed in clinical trials (i.e., ∼1 mg/kg/day). Mechanism-related investigations associated pain-reducing effects with the inhibition of prostaglandin synthesis.

## 6 Epilepsy

**Figure 7:**
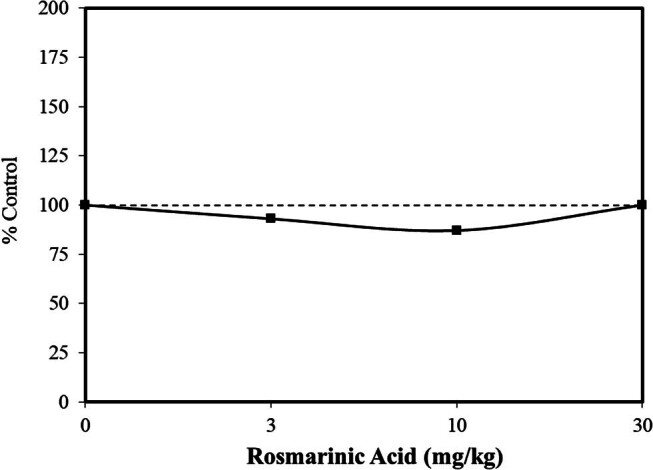
Effects of rosmarinic acid on the generalized seizure incidence in female C57BL/6 mice (modified from Gruigoletto et al. [37]).

An agent with therapeutic potential in the treatment of epilepsy has been typically one that increases the threshold concentration of a seizure-inducing agent, such as pilocarpine, flurothyl, kainic acid, and pentylenetrazol (PTZ). The findings of such studies in animal models are designed to clarify human epileptic responses, including mature seizures [i.e., minimal clonic and generalized tonic-clonic seizures which display initial muscular contraction (i.e., tonic phase) followed by rhythmic muscular contractions (i.e., clonic phase)]. The preclinical data indicate that numerous drugs display proconvulsant and anticonvulsant effects showing hormetic responses, including gamma-aminobutyric acid (GABA) agonists (e.g., muscinol, ascorbate, chloroquine, and 17-beta estradiol) and various opiates (e.g., morphine, fentanyl, pethidine, and others) [30]. Using the same experimental context of the above studies, Gruigoletto et al. [37] reported that rosmarinic acid (i.e., 3, 10, 30 mg/kg – oral, 60 min prior to PTZ treatment) acted as an anticonvulsant agent, preventing seizures in a hormetic manner in the adult female C57BL/6 mouse model (Figure 7). They also showed that the administration of rosmarinic acid within a postconditioning exposure framework reduced generalized pilocarpine-induced seizures over a 14 consecutive day exposure period (3 or 30 mg/kg/day – oral), showing a protective effect at both doses. The protective features of rosmarinic acid were consistent with previous studies using other seizure exposure models [30]. Preliminary mechanistic studies suggested that rosmarinic acid activates the GABAergic system, degrading the enzyme GABA transaminase, resulting in an increase in GABA brain levels.

## 7 Post-traumatic stress disorder (PTSD)

**Figure 8:**
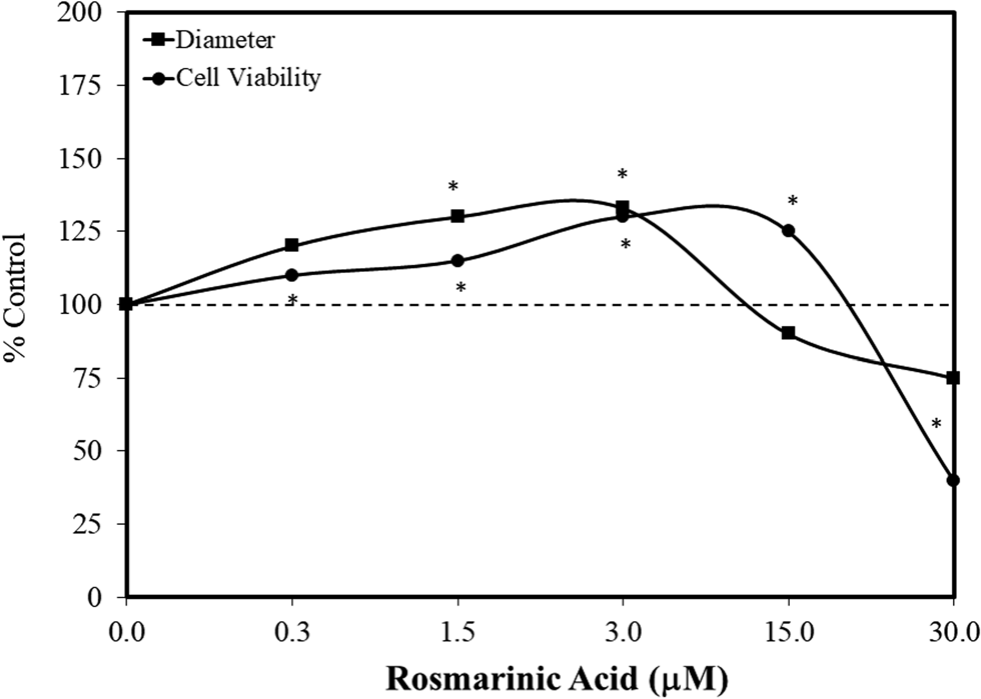
Effects of rosmarinic acid on cultured hippocampal neural stem cells (WST assay) (modified from Nie et al. [39]) (*= statistical significance *P* = <0.05).

Rosmarinic acid enhances cognitive performance and appears to reduce the likelihood and/or severity of a range of neurological pathology, such as seizures, anxiety, and PTSD, among other conditions. Various investigations suggested that it may facilitate positive effects by promoting cell proliferation in the hippocampus [38]. In a follow-up study, Nie et al. [39] obtained hippocampal-derived neural stem cells from embryonic SD rat brains (E 14.5 days), assessing viability via the water soluble tetrasodium salt (WST-1) assay. Neurospheres were also assessed by a broad range of rosmarinic acid concentrations (1–100 µg/ml). Hormetic dose responses were reported for cell viability and spheric diameter (Figure 8) [39]. The use of the ERK1/2 pathway inhibitor, UO106, blocked the stimulatory effects of the optimal concentration for cell viability. A limited experiment using specific cell proliferation assay endpoints at the optimal concentration also showed a similar magnitude of stimulation that was also blocked by the same pathway inhibitor. These findings were significant since the ERK1/2 pathway is essential for cell proliferation, cell differentiation, and cell migration. These findings were related back to *in vivo* experiments in which rosmarinic acid was associated with a significant reduction in PTSD-like symptoms. The rosmarinic acid *in vivo* treatment restored hippocampus proliferation and ERK1/2 expression within 8-week-old rats receiving an IP exposure for 14 consecutive days. These findings linked the capacity of rosmarinic acid to experimentally enhance neural stem cell and hippocampal cell proliferation, establishing a proof of concept. However, the issue of whether rosmarinic acid or its metabolites cross the BBB was not addressed, nor how the observed effects might be mediated.

## 8 Parkinson’s disease and neuroprotection

**Figure 9:**
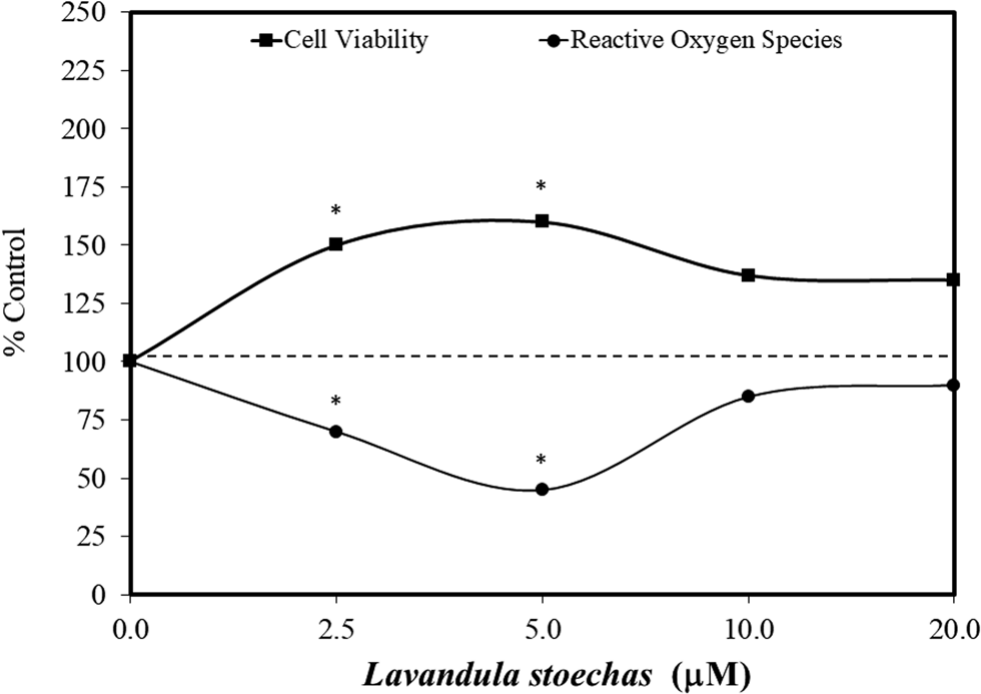
Effects of *Lavandula stoechas* extract (with rosmarinic acid) on PC12 cells (modified from Tayarani-Najaran et al. [40]) (*= statistical significance *P* = <0.05).

The use of hormetic models in therapeutic strategies against neurodegenerative conditions has emerged over the past two decades. Such research has linked hormetic mechanisms principally via the use of experimental preconditioning protocols that modify adaptive mechanisms that in turn, prevent/reduce damage induced by 6-hydroxydopamine (6-OHDA), 1-methyl-4-phenyl-1, 2, 3, 6-tetrahydropyridine, rotenone, or paraquat, using a range of screening approaches based on *in vitro* biological models (e.g., PC12, SH-SY5Y, and MN9 cells) that mimic essential features of Parkinson's disease when exposed to any of the above model toxic agents. Such *in vitro* studies have often involved the use of a broad range of concentrations, facilitating concentration–response evaluations. Within this context, Tayarani-Najaran et al. [40] assessed the effects of *Lavandula stoechas* L. menthol extract against 6-OHDA-induced apoptosis using PC12 cells. Of relevance is that rosmarinic acid is a major component of the *L. stoechas* L. methanol extract. Using a preconditioning 24-h protocol, the *L. stoechas* methanol extract significantly reduced the toxicity of 6-OHDA on cell viability (Alamar Blue assay) (Figure 9). The preconditioning treatment also reduced reactive oxygen species (ROS) production within a hormetic dose–response manner. Similar protective effects of *Lavandula* spp. have been reported in the concurrent treatment of the astrocyte cell line, A172, against hydrogen peroxide (H_2_O_2_)-induced toxicity (i.e., cell viability) [41]. Likewise, rosmarinic acid pretreatment (24 h) protected MES-235 dopaminergic cells by decreasing 6-OHDA-induced ROS production and apoptosis [42].

The mechanism by which 6-OHDA-induced apoptosis in PC12 cells involves activation of the mitogen-activated protein kinase pathway and increasing cleaved poly(ADP-ribose)polymerases. However, the protective pretreatment inhibited the stress-activated protein kinases/Jun amino-terminal kinases (pSAPK/JNK) to SAPK/JNK ratio, reducing apoptosis. Likewise, this pretreatment with *L. stoechas* L. methanol extract enhanced ERK1/2, reducing the cell death pathway activity [40]. This investigative constellation of induced adaptive responses prevented the occurrence of 6-OHDA-induced neural degradation in this Parkinson's disease cellular model.

## 9 Ciguatera fish poisoning (CFP)

CFP is due to eating tropical coral reef fish with elevated concentrations of ciguatera toxins (CTXs). The CTX family of neurotoxins can bioaccumulate within trophic food chains. Their neurotoxicity is due to affinity for site five of the voltage-gated sodium channel VGSC. While mortality due to CTXs is relatively low, about 50,000–100,000 toxicity cases are annually reported with a mixture of neurological and gastrointestinal symptoms [43].

Several plant species have shown chemopreventive potential in different bioassays against CTX-induced toxicity. Of particular interest to the present paper is the potential of *Heliotropium foertherianum,* which contains substantial quantities of rosmarinic acid. In follow-up investigations by Rossi et al. [43], aqueous extracts of *H. foertherianum* leaves and rosmarinic acid blocked the toxicity of CTXs in several bioassays using the neuroblastoma cell model system. Both the leaf extracts and the rosmarinic acid showed hormetic dose responses for cell viability [(3-(4,5-dimethylthiazolyl-2)-2,5-diphenyltetrazolium bromide (MMT assay)], neutral red assay, and the lactate dehydrogenase (LDH) assay, with the maximum stimulatory responses in the 40–50% range. The close quantitative features of the leaf extract and rosmarinic acid bioassay responses suggested that the protective effects of the leaf extract were largely due to the presence of the rosmarinic acid. The authors undertook a detailed structure–activity assessment since several chemical relatives of rosmarinic acid were without treatment effects in these assays. This structure–activity insight may have general utility with application to other endpoints.

## 10 Adipose stem cells

**Figure 10:**
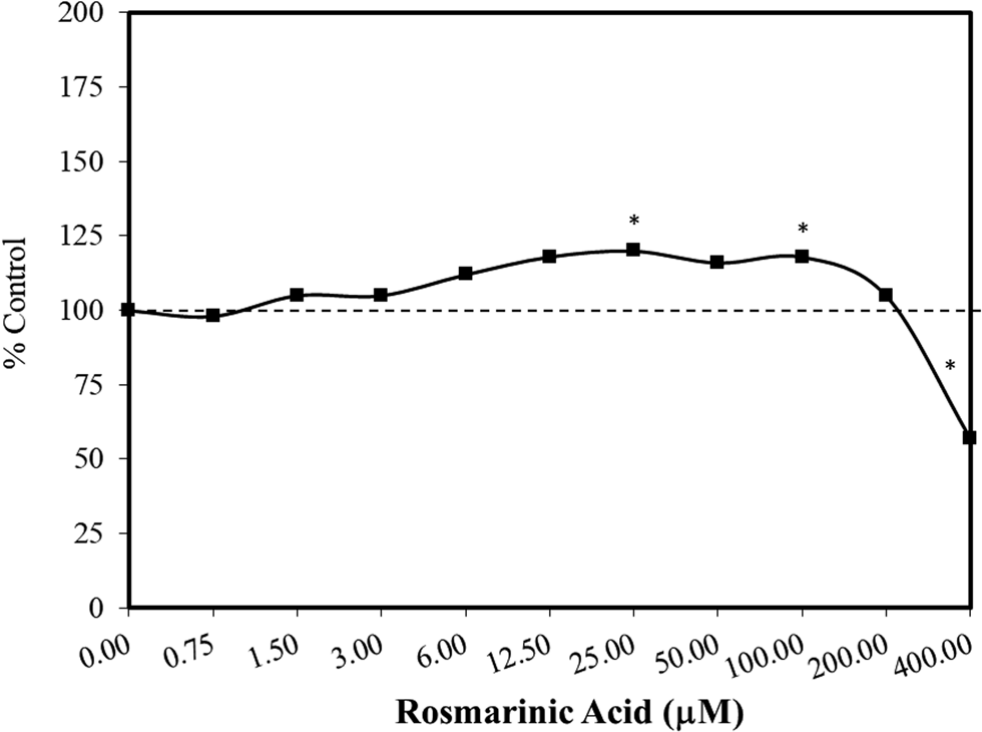
Effects of rosmarinic acid cell viability (MTT assay) on the rat (Wistar adult males) adipose tissue-derived stem cells (modified from Ghorbani et al. [45]) (*= statistical significance *P* = <0.05).

In 2021, Calabrese [44] reported that numerous agents, including various pharmaceuticals, plant-based dietary supplements, and endogenous agents, induced hormetic dose responses in adipose stem cells with a focus on cell proliferation and cell differentiation. Many of the agents inducing hormetic dose responses in adipose stem cells enhanced cell proliferation and cell differentiation at the same concentration when grown in their respective growth media. Based on such studies, Ghorbani et al. [45] assessed the capacity of rosmarinic acid to affect the viability of male Wistar rat adipose stem cells under non-stressed *in vitro* experimental conditions and when under stress from apoptotic factors, such as elevated glucose and serum-deprived conditions. The administration of rosmarinic acid under non-stress conditions induced a hormetic concentration response in the MTT assay (Figure 10) [45]. Rosmarinic acid was then tested in a preconditioning study (4 h) and exposed to either apoptosis-inducing condition. Low concentrations that were non-stimulatory in the non-stressed experiment were protective in the preconditioning studies in a hormetic dose–response manner. Rosmarinic acid also decreased ROS and lipid peroxidation, thereby enhancing survival and reducing apoptotic responses. A similar study by Lin et al. [46] with male SD rat bone marrow cells failed to enhance cell viability in a non-stressed condition while being protective in a preconditioning (2 h) protocol against hydrogen peroxide stress. It is therefore not possible to know whether the difference in response was due to the use of a different stress, a different type of stem cell, or the duration of the preconditioning period.

## 11 Dental pulp stem cells

Apical periodontitis is a serious inflammatory condition affecting the root canal system. This condition is typically adversely affected by microbial contamination and is treated via various antibiotic strategies. However, such treatment approaches have been seen as a type of two-edged sword since they can have an adverse effect on the functioning of dental pulp stem cells that develop into new pulp cells. This situation has led to the exploration of alternative treatment approaches, including the use of rosmarinic acid due to its safety record and antioxidant properties [47]. In a direct head-to-head comparison with leading antibacterial treatments, the rosmarinic acid distinguished itself by inducing a hormetic biphasic dose response, whereas the alternative treatment failed to do so, showing a threshold response followed by toxicity at higher doses. The authors acknowledged the low-dose stimulation by rosmarinic acid, suggesting its potential clinical applications. These findings were conducted within a direct exposure experimental protocol without a companion experiment that involved oxidative or other possible stresses. There is considerable experimental evidence indicating that rosmarinic acid often protects biological systems from various types of oxidative stress within preconditioning-type experimental protocols as shown in this paper. In fact, Andrade et al. [48] reported that rosmarinic acid increased cell viability, reducing phytotoxicity when exposed to a toxic dose of hydrogen peroxide in dental pulp stem cells.

## 12 Muscle

Sarcopenia is a persistent and progressive loss of muscle mass common in many older adults. While it is generally recognized that regular exercise and adequate protein-calorie nutrition can prevent/delay muscle loss, there is considerable interest in finding agents that could safely assist in preventing sarcopenia [49]. Within this context, Lee et al. [50] assessed whether rosemary extract (RE), which contains rosmarinic acid, may be useful in attenuating the development of sarcopenia. They showed that RE enhanced murine myotube differentiation via inhibiting 5-aminoimidazole-4-carboxamide ribonucleoside (AICAR), an AMPK stimulatory agent. The treatment of C2C12 myotube cells with AICAR enhanced the breakdown of protein via the activation of forkhead box transcription factors (FOXO) target genes such as FOXO3a. The RE treatment affected a hormetic dose response for C2C12 cell differentiation.

**Figure 11:**
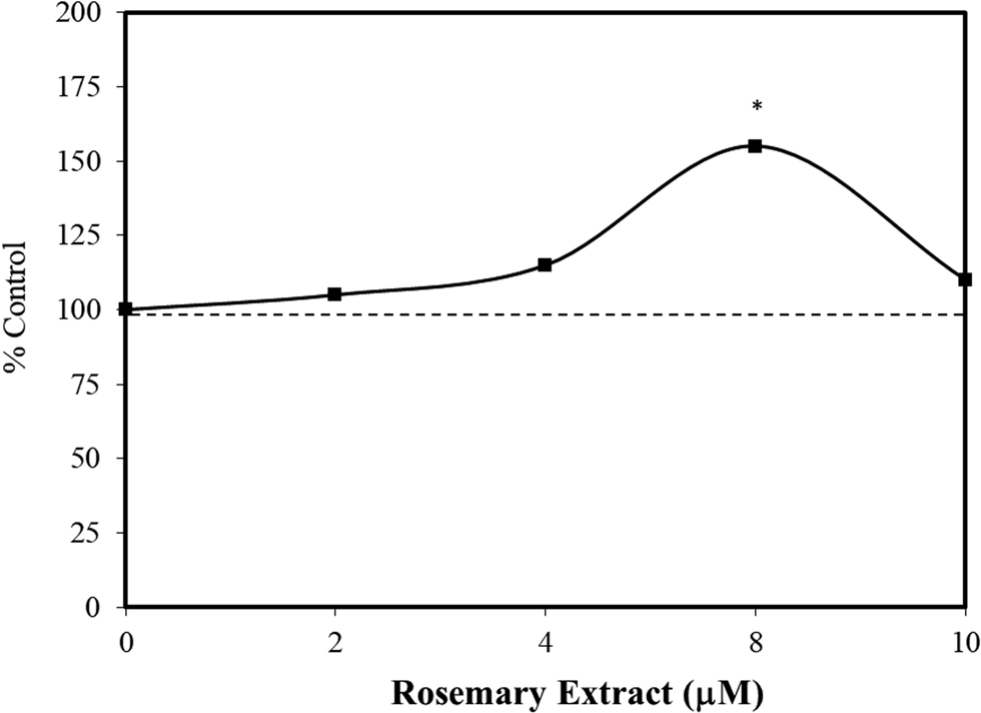
Effects of rosemary extract (RE) on C2C12 myoblast differentiation (modified from Lee et al. [50]) (*= statistical significance *P* = <0.05).

Muscle growth is reduced under conditions such as oxidative stress and nutrient stress, which enhance the activation of AMPK and the subsequent increase in FOXO3a. This process is reversed by the addition of rosemary extract, which inhibits AMPK and FOXO3a nuclear translocation. Such investigations by Lee et al. [50] target ways to slow the process of sarcopenia (Figure 11). The findings of Lee et al. [50] were foreshadowed by an earlier report by Chen et al. [51] that rosmarinic acid not only enhanced the viability of C2C12 cells but also protected these cells from heat stress in a hormetic manner.

## 13 Fibroblasts

**Figure 12:**
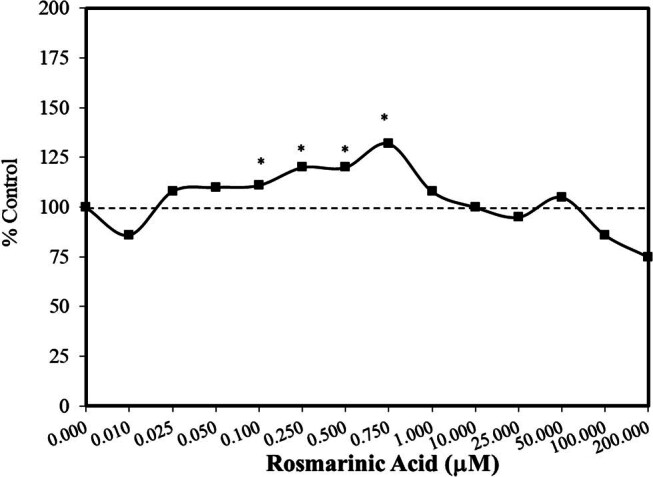
Effect of rosmarinic acid on PCS cells, a normal human skin fibroblast cell strain, using MTT assay (modified from Sodagam et al. [52]) (*= statistical significance *P* = < 0.05).

A research program within the European Union evaluates the effects of phytochemicals on human health for numerous endpoints, such as wound healing. The test agents are initially screened employing a range of experimental models such as the nematode *C. elegans*, along with rats and mice. The first set of chemicals chosen included rosmarinic acid which was evaluated with normal human fibroblasts over a 20,000-fold concentration range employing 13 concentrations [52]. The initial testing showed a low dose stimulation of about 10 to 30% (MTT activity), decreasing at higher concentrations (Figure 12). Despite the low-concentration stimulation, the response at the lowest concentration (0.01 µM) was modestly inhibited. While the reasons for this apparent anomaly were unknown, it was related to unique features of the MTT assay. That is, the MTT assay does not differentiate between cytostatic and cytotoxic effects. The authors were of the opinion that the response at 0.01 µM was not biologically significant because there were no negative effects on the cell growth and proliferation when evaluated over longer periods such as the 7 days of their experiment. In follow-up studies, hormetic stimulatory concentrations protected against ethanol toxicity with rosmarinic acid in a concurrent exposure conditioning protocol.

## 14 Keratinocytes

Since there have been considerable public health and medical concerns with excessive UV exposure causing skin damage and increasing the risk of skin cancer, there is growing interest in assessing the effects of plant-derived compounds that display antioxidant and anti-inflammatory properties [53,54]. Such research interest has been directed to extracts of *Prunella vulgaris* (PV) which has high concentrations of phenolic acids, including rosmarinic acid, which has been used in traditional European and Chinese medicine for its wound healing potential. Based on this background information, Psotova et al. [55] assessed the capacity of PV to protect HaCaT keratinocytes from UV-induced damage. In these post-conditioning studies, the UV was administered first and then followed by PV or rosmarinic acid. In post-conditioning studies, both agents displayed hormetic dose responses for LDH, thiobarturic acid reactive substances (TBARS), and caspase-3. The PV extract was comprised of agents with differing mechanisms of skin protection. In comparison to the PV extract, rosmarinic acid was similar in the level of protection based on LDH and caspase-3 but less so for TBAR. These collective findings indicate that PV and its rosmarinic acid constituent can be protective against UVA-induced damage to HaCaT keratinocytes. More research is needed to clarify the efficacy and degree of protection of PV and rosmarinic acid under varying exposure conditions.

**Figure 13:**
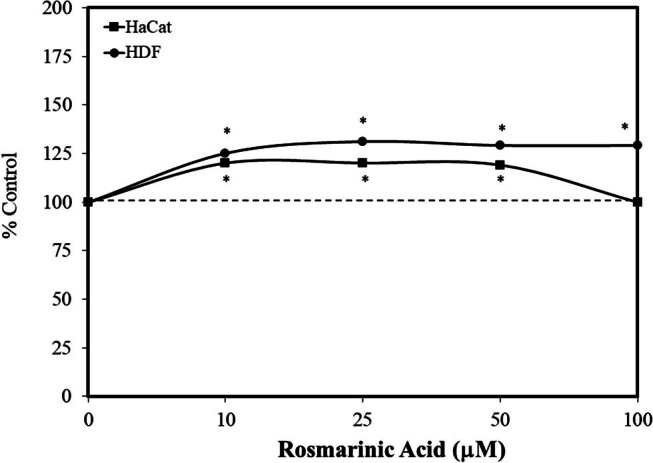
Effects of rosmarinic acid pretreatment (24 h) on the toxicity of ultraviolet B on HDF and human keratinocytes (HaCaT) (MTT assay) (modified from Gupta et al. [56]) (*= statistical significance *P* = < 0.05).

Extending the post-conditioning hormesis study of Psotova et al. [55], Gupta et al. [56] reported that a pretreatment (24 h) with rosmarinic acid enhanced the viability of HaCaT and primary human dermal fibroblasts (HDF) against a subsequent exposure UVB. The UVB treatment reduced cell viability by about 30%, with the rosmarinic acid significantly reversing this toxicity (Figure 13). Rosmarinic acid mediated its protective effects by a restoration of normal mitochondrial function, preventing mitochondria fission due to UVB exposure via the inhibition of UVB-induced Fis-1 expression.

## 15 Cardiomyocytes

Doxorubicin (DOX) is an anthracycline antibiotic that is employed in the treatment of a broad range of cancers in humans. However, its clinical application has been affected by its capacity to cause dose-dependent induction of cardiotoxicity and congestive heart failure. The principal underlying causes of its cardiotoxicity are due to generating ROS, reducing glutathione (GSH), and increasing multiple biomarkers of oxidative stress, which lead to the occurrence of mitochondrial dysfunction, DNA damage, and apoptosis. This situation stimulated interest in finding agents that could significantly reduce DOX-induced cardiotoxicity. One of the agents identified with the potential to block DOX-induced cardiotoxicity is rosmarinic acid, with initial evidence by Psotova et al. [57] with rat cardiomyocytes.

**Figure 14:**
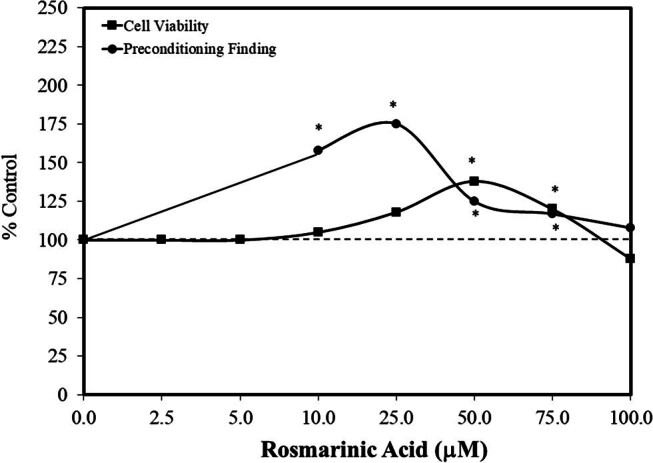
Effects of rosmarinic acid on cell viability in direct exposure and in preconditioning studies to prevent H_2_O_2_ toxicity in MCF-7 cells (modified from Rahbardar et al. [58]) (*= statistical significance *P* = < 0.05).

Of interest is that rosmarinic acid displayed a hormetic dose–response in a direct exposure cell viability study with MCF-7 cells using seven concentrations (2.5–100 µM) [58]. Based on the findings obtained in this direct exposure experiment, they performed a follow-up preconditioning (24 h) experiment using optimal (i.e., stimulatory) concentrations gleaned from the direct activity study (i.e., MCF-7 – MTT assay). Rosmarinic acid induced a hormetic concentration response, with the optimal response returning to control-like values, showing an absolute 40% increase after the initial toxicity of DOX (Figure 14). These findings indicate that rosmarinic acid stimulated growth parameters in a direct-acting experiment, which is primarily an anabolic process. Rosemarinic acid also showed similar stimulatory effects during the preconditioning study when the cells were placed under considerable oxidative stress, switching to a catabolic process. This suggests that rosmarinic acid induced both anabolic and catabolic hormesis.

Rahbardar et al. [58] also included an *in vivo* preconditioning experiment with rats in which the rosmarinic acid treatment reduced the cardiotoxicity effects of DOX based on multiple physiological parameters as well as histopathological endpoints. However, this confirming study employed three rosmarinic doses (10–40 mg/kg/day – IP) for 16 days with DOX treatment starting 4 days later, for a period of 12 days. Another preconditioning study was reported by Quan et al. [59] in which orally administered rosmarinic acid (100 mg/kg) protected mice against the cardiotoxic effects of myocardial ischemia/reperfusion (I/R) injury administered one week later. These collective findings indicate that extremely high doses (oral/IP) of rosmarinic acid have the potential to protect cardiomyocytes from a range of acute toxic insults (e.g., DOX, I/R injury). Future research is needed to assess whether the chemoprotective findings reported in such experimental protocols have translational value.

## 16 Sperm

**Figure 15:**
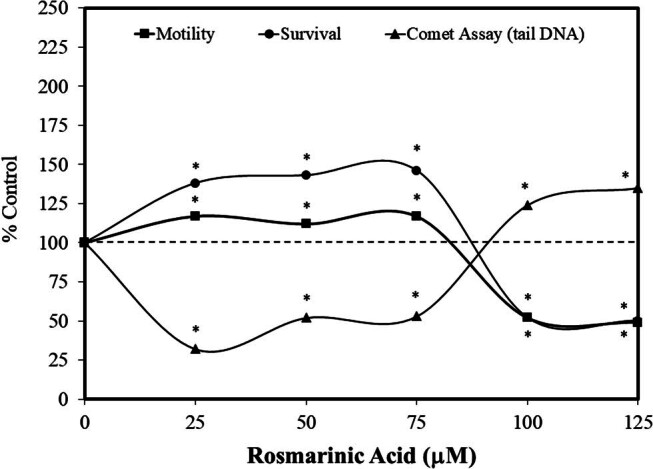
Effects of rosmarinic acid on spotted halibut functional quality during cryopreservation (modified from Zidni et al. [64]) (*= statistical significance *P* = < 0.05).

It is quite common that plant-based dietary supplements are assessed for their capacity to protect sperm during the cryopreservation freezing and thawing process. This scientific interest derives from commercial activities, principally in large animal husbandry and fish farming. In fact, numerous agents have been tested in a comprehensive manner, using remarkably extensive and standard evaluation procedures, typically employing a large number of concentrations, in attempts to identify optimal treatments. Thus, it was not surprising in the assessment of the possible hormetic effects of rosmarinic acid that efforts were made to assess its capacity to protect sperm from the freezing and thawing cryopreservation stress. Such *in vitro* research showed the occurrence of rosmarinic acid-induced hormetic responses on cryopreserved stressed sperm, including Holstein breed bulls [60], Chai rams [61], and Landrace boars [62] and two fish species (i.e., spotted halibut and turbot) [63,64] (Figure 15). These studies typically reported multiple aspects of sperm motility (e.g., curvilinear, straight line, and average path velocities, linear and straightness indexes, oscillation index, amplitude of lateral head displacement, and beat frequency). In addition, experiments were also analyzed for fertilization and hatching rates as well as DNA fragmentation. These studies generally followed the same research strategy with respect to endpoints of interest while using from 4 to 6 concentrations of rosmarinic acid. In each case, a hormetic dose–response pattern was reported for numerous endpoints. The only partial exception was the report of Yeni et al. [60] which failed to show rosmarinic acid-induced improvements in bull sperm motility. However, this study showed the occurrence of DNA damage as well as changes in oxidant generation and antioxidant protection (glutathione peroxidase – GSH_px_) following hormetic dose–response patterns. No mechanistic explanation was provided for the lack of motility enhancing effect in the Yeni et al. [60] study. However, the preservation procedures between the two fish studies were quite different, using different cryopreservation processes (e.g., DMSO versus Ringer solution) as well as other possible significant differences, making the studies difficult to directly compare.

In contrast to its protective effects on boar, ram, and fish sperm, Lv et al. [65] reported that rosmarinic acid not only failed to affect human sperm viability, but it also dose-dependently diminished sperm mobility, penetration, capacitance, and spontaneous acrosomal reaction with a mechanism that involved decreases in calcium via suppression of the potassium channel. While the present findings show that rosmarinic acid is a protective cryopreservation agent for sperm for large animal husbandry and fish-related aquaculture, its role in human sperm biology represents an intriguing and very much open research question.

## 17 Tumor cells

**Figure 16:**
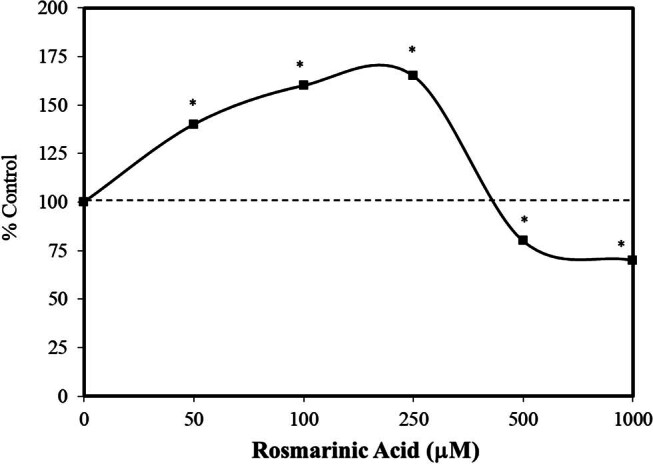
Effects of rosmarinic acid on cell viability of human ovarian carcinoma cells (OVCAR3) (24 h) (modified from Sari and Zaman [69]) (*= statistical significance *P* = < 0.05).

While rosmarinic acid has been widely studied as a possible cancer chemotherapeutic agent for a wide range of tumor types, its capacity to enhance tumor growth at lower concentrations has generally been deemphasized relative to its capacity for tumor inhibition. Nonetheless, lower concentrations of rosmarinic acid enhance cell proliferation in a broad range of tumor cells such as A 549 cells (lung) [66], HCT-116 (colorectal) [66], multiple types of breast cancers and MCF-7 and MCF-10A [66,67], prostate (Du-145; PC-3) [68], human chronic myeloid leukemia [68], ovarian adenocarcinoma carcinoma (OVCAR3) (Figure 16) [69], and C6 cells (glioblastoma cells) [70]. It is important to note that the quantitative features of the hormetic dose response for tumor cell lines display the same quantitative constraints as seen for non-tumor cells for cell proliferation [71]. Importantly, possible low dose stimulation of tumor cells by numerous agents, especially plant-based dietary supplements, remains to be more fully explored and assessed with respect to its biomedical implications. However, the lower tumor cell stimulatory concentrations reported for rosmarinic acid are far greater than would be experienced via the normal range of human exposures, making it an unrealistic occurrence/risk.

## 18 Lifespan

Extending the health span and lifespan has become a topic of major interest and a widespread research priority. Numerous possible interventions have been reported in experimental models using various pharmaceutical agents, plant-based dietary supplements, lifestyle activities (e.g., exercise), stress management strategies, and the use of saunas, cold showers, and other approaches. While numerous biological models have been used in these evaluations, the most widely used model in recent years has been the nematode *C. elegans*, principally based on its size, lifespan, and genetic characteristics. The collective evidence indicates that health span and lifespan can be modestly, but significantly, enhanced in multiple experimental studies, with many showing that these increases display hormetic dose responses for both health span and lifespan. These studies have collectively shown that the median increase in lifespan is modest being in the 20–30% range [72,73]. Pietsch et al. [5] have also reported that rosmarinic acid extended the lifespan of *C. elegans* in a statistically significant manner. Genetic mutant investigations by these researchers linked lifespan extension to specific genes and their affected metabolic pathways. These investigations suggested that the lifespan extension was associated with the activation of antioxidant properties, a characteristic that is common across a broad range of similar lifespan-extending agents.

## 19 Mutation

**Figure 17:**
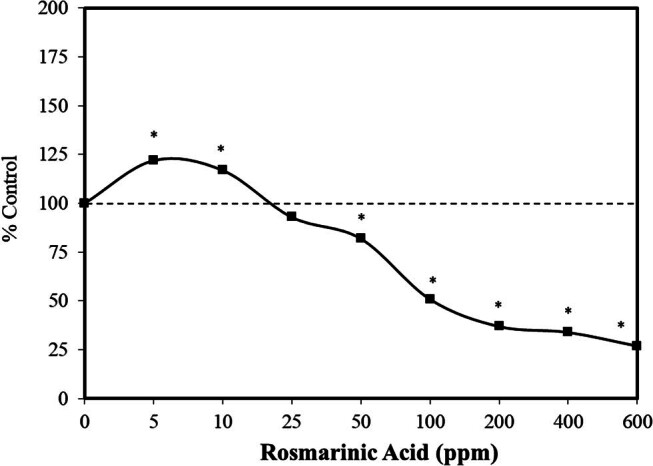
Effects of rosmarinic acid on allium root growth (i.e., root length) (modified from Liman et al. [74]) (*= statistical significance *P* = < 0.05).

Rosmarinic acid has been evaluated in numerous animal experimental systems for a vast range of biological functions and endpoints. While not commonly tested for its toxicological effects on plant systems, Liman et al. [74] evaluated its cytotoxic and genotoxic effects on *Allium cepa* L. root meristem cells. This study revealed that rosmarinic acid adversely affects plant cells at very high concentrations (e.g., DNA damage and chromosomal aberrations). However, they also assessed its capacity to affect allium root growth across a broad concentration range (5–600 ppm). The rosmarinic acid increased, in a hormetic manner, growth as measured by root length (Figure 17). The low doses were not damaging to the chromosome or DNA. While the focus of the Liman et al. [74] research was principally on its genetic toxicity at high concentrations, the striking hormetic plant growth responses are of biological interest and worthy of further research.

## 20 Liver: Hepatic stellate cell

**Figure 18:**
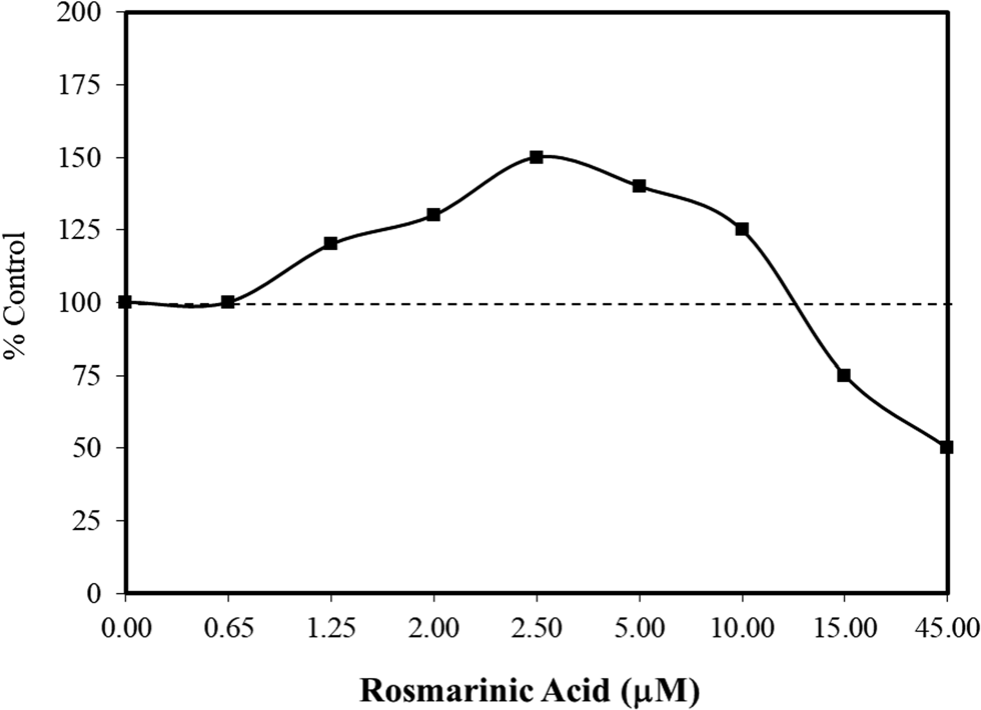
Effects of rosmarinic acid on the cell proliferation of CCC-HEL (hepatic stellate cells) (modified from Zhang et al. [75]).

Hepatic fibrosis occurs principally as a byproduct of the wound-healing process following repeated injury to the liver. During this process of wound healing, normally quiescent hepatic stellate cells (HSC) become highly activated and display a broad spectrum of physiological and morphological changes, ultimately becoming a central component of pathologies relating to the fibrosis process. Clinical studies for treating this condition involve the targeting of cells to prevent cell proliferation and enhance apoptosis. Using CCC HEL cells, it was shown that rosmarinic acid biphasically affected cell proliferation, increasing at low concentrations while enhancing apoptosis at higher concentrations (Figure 18) [75]. These findings were subsequently extended by El-Lakkany et al. [76] using rat HSC-T6 cells. They reported that the rosmarinic acid displayed a dose-dependent decrease in cell survival using the SRB (sulforhodamine B dye) approach at 24 h. However, by 48 h there was a compensatory response for cell survival, increasing by approximately 35% at the lower concentration. Higher concentrations of rosmarinic acid affected an increase in apoptosis. Thus, in the case of liver fibrosis, the rosmarinic acid can enhance or inhibit the process, depending on the dosage, with the lower dose stimulatory effects enhancing pathological processes.

## 21 Liver: Antioxidant enhancement

There have been efforts to improve broiler (chickens bred and raised for meat) growth performance via the use of dietary supplements. Because of its anti-inflammatory properties and its capacity to scavenge free radicals, it has been suggested that rosmarinic acid supplementation may have practical utility for enhancing the growth and overall health of broilers. While various studies with limited study designs (i.e., very few doses) have supported the potential of rosmarinic acid to enhance broiler growth performance, Shang et al. [77] reported that rosmarinic acid enhanced the liver antioxidant status of broilers via its addition to the diet. Both superoxide dismutase and catalase activities were enhanced, showing a hormetic biphasic dose response. In a similar manner, the rosmarinic acid decreased malondialdehyde levels, showing a U-shaped dose response. Thus, these studies identified an optimal range for rosmarinic acid supplementation. In addition to the hepatic enhancement of antioxidant enzymes, the flavor of the muscle meat was also improved by the supplementation of rosmarinic acid at 200 mg/kg which was the optimal concentration for oxidative enzyme effects as well. This improvement was most strongly related to a 17% increase in glutamic acid in muscle tissue at the optimum dosage. This increase in the glutamic acid biomarker provided the principal basis for the conclusion that meat quality and flavor had been enhanced by the rosmarinic acid treatment.

## 22 Oocyte maturation

Numerous reports in the scientific literature indicate that dietary, endogenous, and other agents induce hormetic responses in oocytes, their supportive cells (e.g., granulosa cells), blastocyst formation, and early-stage embryonic development. Such studies have further shown that numerous agents enhance oocyte-related biological functioning under normal conditions as well as enhancing its capacity to prevent damage from chemical toxins and related oxidant stressors (e.g., heat and age-related processes) in preconditioning and concurrent exposures. Within this context, Zhang et al. [78] assessed the effects of rosmarinic acid during oocyte maturation, development, and hatching ability after parthenogenic activation. These authors reported that rosmarinic acid during such *in vitro* maturation studies enhanced the hatching rate as measured by polar body extrusion by 22%. These findings suggest that rosmarinic acid may enhance the developmental competence of porcine oocytes during the *in vitro* maturation process by reducing oxidative stress.

## 23 Discussion

Rosmarinic acid has attracted considerable clinical research interest, being the subject of multiple clinical trials for a broad range of biomedical/therapeutic indications, including cognitive functions, pain, and nasal polyp inflammation with dietary exposures of 500–900 mg/day [2,36,79,80]. While the results of such clinical trials have been encouraging, the underlying foundations that have supported the trials were largely derived from *in vitro* and *in vivo* animal model studies, often suggesting hormetic dose–response findings as discussed herein.

Of importance in the evaluation of rosmarinic acid are its bioavailability, metabolism, and tissue distribution (including the capacity to affect and/or cross the BBB and its distribution and half-life within the brain). Several studies have assessed the GI tract absorption involving adult male SD and Wistar rats [81,82,83,84], humans [79,85,86], via *in silico* calculations [87] and using mechanistically based approaches via enterocytes by paracellular transport in tight junctions [80,88,89].

Gastrointestinal tract bioavailability with adult male SD rats showed 31.8% absorption of rosmarinic acid (200 mg/kg with food withdrawn at 18 h prior to exposure) over a 48-h time period based on urinary measurements of rosmarinic acid and six metabolites [81]. Rosmarinic acid metabolites were not found in the bile, supporting the conclusion that urinary excretion was dominant. The authors suggested that gut flora was the likely cause of metabolic transformation and subsequent GI tract absorption, supporting the earlier findings of Goodwin et al. [90]. By 12 h, only 6.2% of the administered dose had been excreted. By 36 h, the excretion had achieved 30.4%. Follow-up research by Baba et al. [85] with male SD rats was consistent with the findings of Nakazawa and Ohsawa [81]. Thus, the rosmarinic acid GI tract absorption data reported in rodents, though highly consistent, are quite limited, involving two studies with only 12 young male SD rats.

Several human GI tract absorption studies have been published [79,85,86,90], but only Baba et al. [85] and Noguchi-Schinohara [91] provided quantification based on young healthy adults (37-year-old average) given a 200- or 500-mg dose, respectively. The GI tract absorption in the Baba et al. [85] study over 48 h was 6.3%, with 75% being excreted in the urine by 6 h. These authors suggested that the rosmarinic acid bioavailability was significantly affected by its metabolism from microbial esterases in the GI tract, hydrolyzing the ester link in rosmarinic acid with only limited P-dehydroxylation of the caffeic acid metabolite. The caffeic acid derived from rosmarinic acid is then absorbed, conjugated, and methylated in the digestive system and the liver. In the case of the Noguchi-Shinohara et al. [91] study, the results were similar, with the low peak serum rosmarinic acid levels (<0.5 µM) being seen after 1 h in fasted subjects.

The question of whether rosmarinic acid reaches the brain following oral, IP, or IV administration has been explored [84,92,93]. In the study of Chen et al. [84], rats were administered the rosmarinic acid intragastrically (600 mg/kg), which is equivalent to about 150 µmol/kg rosmarinic acid or via IP at 1,000 mg/kg, which is equivalent to about 250 µmol/kg. The dosing strategy was designed to achieve high levels of different metabolites for analytical quantification purposes. These approaches were similarly followed by others [89]. Regardless of the route of administration, dosing, and animal model used, the capacity for rosmarinic acid to pass the BBB is very limited at best. While it did not pass the BBB when the exposure was oral, rosmarinic acid was detected at very low levels in the brain when given IP or IV.

These observations suggested that rosmarinic acid may have effects on the brain but via an indirect mechanism. For example, Hase et al. [93] suggested that rosmarinic acid effects may be mediated by a brain-gut axis, i.e., signals to the brain via complex vagal linkage between the small intestine and the brain. They further hypothesized that this may be how rosmarinic acid affected increases in monoamine levels (e.g., dopamine, levodopa, norepinephrine) since high levels of rosmarinic acid may exist in the gut after the feeding.

Since rosmarinic acid affected a variety of brain related neurological effects during *in vivo* studies, there has been interest in assessing the mechanism as an extension to the above bioavailability and tissue distribution studies. A consideration of these studies highlights the uncertainty concerning how rosmarinic acid affected these behavioral/neuroprotective responses. In the case of Park et al. [27], they stated that “it is likely that rosmarinic acid penetrates the brain and exerts its biological activities including cognition enhancing properties although its amount is low” (page 646 right column bottom). In the case of seizures, Gruigoletto et al. [37] hypothesized that rosmarinic acid may activate the GABAergic system based on *in vitro* experiments. In the case of pain, Boonyarikpunchai et al. [36] suggested that rosmarinic acid-mediated these responses by acting both centrally and peripherally. Ito et al. [38] showed that the IP administration of rosmarinic acid enhanced cell proliferation of the hippocampus cells. They stated that “it is still unclear whether rosmarinic acid modulates the increase in cell proliferation directly or indirectly although rosmarinic acid can be translocated to the brain,” citing Li et al., [83]. They further stated that “our preliminary data denotes that rosmarinic acid did not directly exhibit a proliferative effect on rat fetal brain derived neural stem propagation cells *in vitro*.” Thus, rosmarinic acid may indirectly rather than directly affect cell proliferation within the dentate gyrus. In fact, Li et al. [83] stated that rosmarinic acid does not efficiently cross the BBB, repeating this conclusion later in the article for emphasis. In contrast to uncertainty over rosmarinic acid-induced mechanisms in the above-cited articles, it induced protection against traumatic brain damage in a rat model, which was due, in large part, to its capacity to enhance the integrity of the BBB [94].

In addition to *in vivo* rosmarinic acid-based behavioral/neuroprotective studies [33,36,37] that used very high oral doses whose results are difficult to extrapolate to humans, several groups [31,32,38] used IP exposures that resulted in quantities of rosmarinic acid that were largely unachievable via oral exposures creating very unrealistic human exposure scenarios. In contrast, the Park et al. [27] study concerning cognitive performance was based on relatively realistic oral human dosing using the rat model. However, the bioavailability in the rat exceeds that of humans by about 5 to 6-fold, diminishing, to some extent, the practical relevance of this study for human application. In addition, this study also failed to provide the number of animals in the control and treatment groups, affecting the capacity for study evaluation.

In light of the above consideration, it is necessary to assess pharmacokinetic features of dietary polyphenols, such as rosmarinic acid, along with oral pharmaceuticals. As discussed by Kroon et al. [95], a large proportion of drugs are designed/used to be metabolized relatively slowly. Furthermore, they are typically ingested at dosages sufficiently high that most of the administered dose escapes first-pass liver metabolism. This results in a sufficient quantity of the active agent (in the unmetabolized unconjugated form) for distribution to the appropriate tissues. However, in the case of polyphenols, including rosmarinic acid, they are typically delivered at low doses in the diet and, in most instances, they do not escape first-pass metabolism, with the prominent chemical forms being conjugates of glucuronides and sulfates, with or without methylation. These conjugated metabolites are chemically distinct from the parent compound, showing considerable differences in size, polarity, and ionic form. Thus, their biological actions are quite different from the parent compound. Consequently, Kroon et al. [95] concluded that *in vitro* evaluations of polyphenols for chemoprotection purposes need to use appropriate conjugates as found *in vivo*. In addition, bioavailability studies reveal that maximum concentrations in plasma typically do not exceed 1 µM following the consumption of 10–100 mg of a single phenolic compound, with the maximum concentration occurring typically less than 2 h after ingestion, then dropping quickly thereafter. Kroon et al. [95] concluded that “we strongly recommend that all experiments using *in vitro* models to study biological responses to dietary polyphenols use only physiologically relevant flavonoids and their conjugates at appropriate concentrations, provide evidence to support their use, and justify any conclusions generated. When authors fail to do this, referees and editors must act to ensure that data obtained *in vitro* are relevant to what might occur *in vivo*,” a perspective supported more than a decade later by Gonzales [96], suggesting that this concern had not been adequately addressed at the level of researcher, reviewer, and editor.

In the case of the *in vitro* studies assessed herein, and with few exceptions, most of the studies employed concentrations >10 µM with some studies involving concentrations in the several hundred µM range, with the duration of the exposure typically in the range of 24–72 h, far longer duration than the very short time interval of a few minutes to several hours in human *in vivo* situations. For example, the preconditioning studies as reported for cardiomyocytes [58] and PC12 cells [40] were based on a 24-h period which far exceeds the expected *in vivo* exposure time. However, in the case of preconditioning studies reported here with rosmarinic acid for adipose stem cells, the preconditioning period ranged from 2 to 4 h, depending upon the study, reflecting a more realistic durational exposure [45,46].

There are thus three major concerns in addressing the biological realism of *in vitro* studies of polyphenols, including rosmarinic acid. These include the need to test the most appropriate chemical form and to do testing at realistic concentrations and appropriate durations. In the case of rosmarinic acid, most of the *in vitro* studies reported herein would fail a biological relevance evaluation for each of these three areas of concern. While it is of considerable biological interest to challenge cells during *in vitro* studies to assess the adaptive response-toxicity continuum and their underlying mechanisms, these findings need to be placed in biological perspective. However, it was common in the rosmarinic acid studies assessed that just the opposite occurred; that is, speculation on human applications tended to be offered while ignoring the capacity of the experimental protocols to address actual human exposures. This perspective is not only applicable to rosmarinic acid findings but to most polyphenols as well. When such papers are not placed within the proper context, they may be widely used by commercial enterprises to inappropriately overstate commercial applications for human consumption such as supplement or other uses. While the above comments are directed toward *in vivo* applications of *in vitro* studies, these perspectives are not relevant to sperm cryopreservation studies.

Despite the legitimate concerns raised by Kroon et al. [95], the alternative indirect brain mechanism(s) suggested by Hase et al. [93] need further consideration. A recent paper by Scuto et al. [97] supports the vagus nerve–brain–gut hypothesis of Hase et al. [93] by showing the occurrence of communication between intestinal micro-organisms and the CNS via the gut-brain axis. Scuto et al. [97] speculated that various hormesis-acting phytochemicals (e.g., blueberry extracts, curcumin, resveratrol) stimulate the vagus nerve, effectively modulating microbiota-brain communication strategies with potential for therapeutic applications for protection against pathobiological processes. The actual clinical significance of these perspectives remains to be explored.

The vagus nerve is the longest nerve in the body and provides both afferent and efferent pathways between the gut and brain. It is now seen as a neurometabolic sensor, detecting microbiota metabolites via its afferent fibers, passing this information from the gut to the brain, where it is integrated into the central autonomic network to generate a spectrum of adaptive responses in both the brain and in the intestine. The vagus nerve possesses the capacity to sense and monitor the so-called chemical milieu of the gut via the interaction of nutrients and various gut peptides with afferent fibers modulating and optimizing neurological and gastrointestinal health. Scuto et al. [97] specifically related these biological properties to the actions of a broad range of hormesis-acting polyphenols, proposing that these nutrients act via the vagus nerve to mediate both brain and gut health via hormetic mechanisms. This perspective illustrates how dietary hormetic agents may affect the brain, heart, and the GI tract at biologically “realistic” doses delivered to the GI tract. The perspectives offered by Scuto et al. [97] have the potential to refocus mechanistic research on polyphenolic agents, such as rosmarinic acid, that induce a spectrum of potentially beneficial effects via novel mechanisms at doses that can be achieved via normal dietary and supplement strategies. The necessary caveat is that health claims must be tempered for any indication by demonstration of safety, and clinical as well as statistical significance.

## 24 Conclusion and perspective

The findings of this assessment are significant in two major directions. The first is that the extensive documentation of hormetic dose responses for rosmarinic acid extends the generality of the hormesis concept. These findings support the conclusion that hormesis is an evolutionary-based and highly generalizable strategy for biological systems to manage and incorporate both anabolic (i.e., growth) and catabolic (i.e., protection/defense) functions, showing the centrality of the hormesis concept. The second major direction is that this paper provides the first integrative assessment of hormetic effects induced by rosmarinic acid and its biomedical and therapeutic applications. These findings provide an important foundation in the formulation of future research directions for rosmarinic acid, as well as framing an experimental design strategy to optimize the evaluation of hormetic dose response hypotheses within a rigorous and mechanistically oriented manner.

## References

[1] An Y, Zhao J, Zhang Y, Wu W, Hu J, Hao H, et al. Rosmarinic acid induces proliferation suspension of hepatoma cells associated with NF-ĸB signaling pathway. Asian Pac J Cancer Prev. 2021;22:1623–31. 10.31557/APJCP.2021.22.5.1623. PMC8408391 34048194

[2] Noor S, Muhannad T, Rub MA, Raza A, Azum N, Yadav DK, et al. Biomedical features and therapeutic potential of rosmarinic acid. Arch Pharm Res. 2022;45:205–28. 10.1007/s12272-022-01378-2 PMC8989115 35391712

[3] Ravaria P, Saxena P, Laksmi S, Ranjan V, Abidi SWF, Saha P, et al. Molecular mechanisms of neuroprotective offerings by rosmarinic acid against neurodegenerative and other CNS pathologies. Phytother Res. 2023;37:2119–43. 10.1002/ptr.7825 37014255

[4] Kopp-Schneider A, Lutz WK. J-shaped dose-response relationship for tumor induction by caffeic acid in the rat forestomach, modeled by non-monotonic dose response for DNA damage and cell proliferation. Hum Ecol Risk Assessment. 2001;7:921–31.

[5] Pietsch K, Saul N, Chakrabarti S, Sturzenbaum SR, Menzel R, Steinberg CEW. Formations, antioxidants and pro oxidants: defining curtain, caffeic acid and rosmarinic acid mediated life extension in C. elegans. Biogerontology. 2011;1-2:329–47. 10.1007/s10522-011-9334-7 21503726

[6] Jodynis-Liebert J, Kujawska M. Biphasic dose-response induced by phytochemicals: Experimental Evidence. J Clin Med. 2020;9:718. 10.3390/jcm9030718 PMC7141213 32155852

[7] Murakami A. Impact of hormesis to deepen our understanding of the mechanisms underlying the bioactivities of polyphenols. Curr Opin Biotech. 2024;86:103074. 10.1016/j.copbio.2024.103074 38325232

[8] Calabrese EJ. Neuroscience and hormesis: Overview and general findings. Crit Rev Toxicol. 2008;38:249–52. 10.1080/10408440801981957 18432418

[9] Calabrese EJ. Hormesis: Why it is important to toxicology and toxicologists. Environ Toxicol Chem. 2008;27:1451–74. 10.1897/07-541 18275256

[10] Calabrese EJ. Toxicology rewrites its history and rethinks its future: Giving equal focus to both harmful and beneficial effects. Environ Toxicol Chem. 2011;30:2658–73. 10.1002/etc.687 21932295

[11] Calabrese EJ. The dose-response: A fundamental concept in toxicology. In: Hayes AW, Kruger CL, editors. Principles and methods of toxicology. 6th edn Boca Raton, FL: CRC Press; 2014. p. 89–140.

[12] Calabrese EJ, Baldwin LA. Defining hormesis. Hum Exp Toxicol. 2002;21:91–7. 10.1191/0960327102ht217oa 12102503

[13] Calabrese EJ, Blain R. The occurrence of hormetic dose responses in the toxicological literature, the hormesis database: An overview. Toxicol Appl Pharm. 2005;202:289–301. 10.1016/j.taap.2004.06.023 15667834

[14] Calabrese EJ, Blain RB. Hormesis and plant biology. Environ Poll. 2009;157:42–8. 10.1016/j.envpol.2008.07.028 18790554

[15] Calabrese EJ, Blain RB. The hormesis database: The occurrence of hormetic dose responses in the toxicological literature. Reg Toxicol Pharm. 2011;61:73–81. 10.1016/j.yrtph.2011.06.003 21699952

[16] Calabrese EJ. Evidence that hormesis represents an “overcompensation” response to a disruption in homeostasis. Ecotox Environ Safety. 1999;42:135–7. 10.1006/eesa.1998.1729 10051361

[17] Calabrese EJ. Preconditioning is hormesis part I: Documentation, dose-response features and mechanistic foundations. Pharm Res. 2016;110:242–64. 10.1016/j.phrs.2015.12.021 26757428

[18] Calabrese EJ. Preconditioning is hormesis part II: How the conditioning dose mediates protection: Dose optimization within temporal and mechanistic frameworks. Pharm Res. 2016;110:265–75. 10.1016/j.phrs.2015.12.020 26748033

[19] Calabrese EJ, Baldwin LA. Chemical hormesis: Its historical foundations as a biological hypothesis. Hum Exp Toxicol. 2000;19:2–31. 10.1191/096032700678815585 10745292

[20] Calabrese EJ, Baldwin LA. The marginalization of hormesis. Hum Exp Toxicol. 2000;19:32–40. 10.1191/096032700678815594 10745293

[21] Calabrese EJ, Baldwin LA. Radiation hormesis: Its historical foundations as a biological hypothesis. Hum Exp Toxicol. 2000;19:41–75. 10.1191/096032700678815602 10745294

[22] Calabrese EJ, Baldwin LA. Radiation hormesis: The demise of a legitimate hypothesis. Hum Exp Toxicol. 2000;19:76–84. 10.1191/096032700678815611 10745295

[23] Calabrese EJ, Baldwin LA. Tales of two similar hypotheses: The rise and fall of chemical and radiation hormesis. Hum Exp Toxicol. 2000;19:85–97. 10.1191/096032700678815620 10745296

[24] Calabrese EJ. Hormetic mechanisms. Crit Rev Toxicol. 2013;43:580–606. 10.3109/10408444.2013.808172 23875765

[25] Calabrese EJ, Kozumbo WJ. The hormetic dose-response mechanism: Nrf2 activation. Pharm Res. 2021;167:105526. 10.1016/j.phrs.2021.105526 33667690

[26] Calabrese EJ, Mattson MP. Hormesis provides a generalized quantitative estimate of biological plasticity. J Cell Comm Sign. 2011;5:25–38. 10.1007/s12079-011-0119-1 PMC3058190 21484586

[27] Park DH, Park SJ, Kim JM, Jung WY, Ryu JH. Sub chronic administration of rosmarinic acid, a natural prolyl oligopeptidase inhibitor, enhances cognitive performances. Fitoterapia. 2010;81:644–8. 10.1016/j.fitote.2010.03.010 20230877

[28] Braida D, Paladini E, Griffini P, Lamperti M, Maggi A, Sala M. An adverted U-shaped curve for heptylphysostigmine on radial maze performance in rats: Comparison with other cholinesterase inhibitors. Eur J Pharmacol. 1996;302:13–20. 10.1016/0014-2999(96)00072-6 8790986

[29] Calabrese EJ. Alzheimer’s disease drugs: an application of the hormetic dose response model. Crit Rev Toxicol. 2008;38:419–51. 10.1080/10408440802003991 18568864

[30] Calabrese EJ. Pain and U-shaped dose response: Occurrence mechanisms and clinical implications. Crit Rev Toxicol. 2008;38:579–90. 10.1080/10408440802026281 18709566

[31] Takeda H, Tsuji M, Inazu M, Egashira T, Matsumiya T. Rosmarinic acid and caffeic acid produce antidepressive like effect in the forced swimming test in mice. Eur J Pharm. 2002;449:261–7. 10.1016/s0014-2999(02)02037-x 12167468

[32] Takeda H, Tsuji M, Miyamoto J. Rosmarinic acid and caffeic acid reduce the defensive freezing behavior of mice exposed to conditioned fear stress. Psychopharmacology. 2002;164:233–5. 10.1007/s00213-002-1253-5 12404088

[33] Yousuf S, Sarfaraz Y, Emad S, Qadeer S, Perveen T. Dose dependent effects of lemon peel oil on oxidative stress and psychological behaviors in rats. Pakistan J Zool. 2023;55:1627–35.

[34] Calabrese EJ. Modulation of the epileptic seizure threshold: Implications of biphasic dose responses. Crit Rev Toxicol. 2008;38:543–56. 10.1080/10408440802014261 18615309

[35] Sun HY, Calabrese EJ, Lin Z, Lian B, Zhang XX. Similarities between the Yin/Yang doctrine and hormesis in toxicology and pharmacology. Trends Pharmacol Sci. 2020;41:544–56. 10.1016/j.tips.2020.05.004 PMC7302776 32564900

[36] Boonyarikpunchai W, Sukrong S, Towiwat P. Antinociceptive and anti-inflammatory effects of rosmarinic acid isolated from Thunbergia laurifolia Lindl. Pharm Biochem Behav. 2014;124:67–73. 10.1016/j.pbb.2014.05.004 24836183

[37] Gruigoletto J, de Oliveira CV, Grauncke ACB, de Souza TL, Souto NS, de Freitas ML, et al. Rosmarinic acid is an anticonvulsant against seizures induced by pentylenetetrazol and pilocarpine in mice. Epil Behav. 2016;62:27–34. 10.1016/j.yebeh.2016.06.037 27448240

[38] Ito N, Yabe T, Gamo Y, Nagai T, Oikawa T, Yamada H. Rosmarinic acid from Perillae Herba produces an antidepressant-like effect in mice through cell proliferation in the hippocampus. Biol Pharm Bull. 2008;31:1376–80. 10.1248/bpb.31.1376 18591778

[39] Nie H, Peng Z, Lao N, Wang H, Chen Y, Fang Z, et al. Rosmarinic acid ameliorates PTSD like symptoms in a rat model and promotes cell proliferation in the hippocampus. Prog Neuro-Psychopharm Biol Psychol. 2014;51:16–22. 10.1016/j.pnpbp.2014.01.002 24418162

[40] Tayarani-Najaran Z, Hadipour E, Mousavi SMS, Emami SA, Mohtashami L, Javadi B. Protective effects of Lavandula stoechus L. methanol extract against 6-ODHA induced apoptosis in PC12 cells. J Ethnopharm. 2021;273:114023. 10.1016/j.jep.2021.114023 33716081

[41] Costa P, Sarmento B, Goncalves B, Romano A. Protective effects of Lavandula viridis L’Her extract and rosmarinic acid against H2O2-induced oxidative damage in A172 human astrocyte cell line. Indus Crop Prod. 2013;50:361–5.

[42] Ren P, Jiang H, Li R, Wang J, Song N, Xu H-M, et al. Rosmarinic acid inhibits 6-OHDA-induced neurotoxicity by anti-oxidation and MES23.5 cells. J Mol Neurosci. 2009;39:220–5. 10.1007/s12031-009-9182-y 19219567

[43] Rossi F, Jullian V, Pawlowiez R, Kumar-Roine S, Haddad M, Darius HT, et al. Protective effect of Heliotropium foetherianum (Boraginacease) folk remedy and its active compound, rosmarinic acid, against a Pacific ciguatoxin. J Ethnopharm. 2012;143:33–40. 10.1016/j.jep.2012.05.045 22706150

[44] Calabrese EJ. Hormesis and adult adipose-derived stem cells. Pharm Res. 2021;172:105802. 10.1016/j.phrs.2021.105803 34364988

[45] Ghorbani A, Sadeghnia HR, Afshari AR, Hosseimi A. Rosmarinic acid protects adipose tissue-derived mesenchymal stem cells in nutrient-deficient conditions. Prev Nutr Food Sci. 2019;24:449–55. 10.3746/pnf.2019.24.4.449 PMC6941722 31915641

[46] Lin LZ, Chen HH, Lei ZX, Li YR, Zhou CH, Huang YC, et al. Rosmarinic acid protects on rat bone marrow mesenchymal stem cells from hydrogen peroxide-induced apoptosis. J Asian Nat Prod Res. 2018;20:570–80. 10.1080/10286020.2018.1426571 29376419

[47] Othman NM, Elhawary YM, Elbeltagy MG, Badr AE. The effect of Rosmarinus officialis as a potential root canal medication on the viability of dental pulp stem cells. J Contem Dent Prac. 2023;24:623–31. 10.5005/jp-journals-10024-3570 38152933

[48] Andrade JMM, Maurmann N, Lopes DV, Pereira DP, Pranke P, Henriques AT. Rosmarinic acid and chlorogenic acid, isolated from ferns, suppressed stem cell damage induced by hydrogen peroxide. J Pharm Pharmacol. 2022;74:1609–17. 10.1093/jpp/rgac061 36029199

[49] Calabrese EJ, Calabrese V. Enhancing health span: Muscle stem cells and hormesis. Biogerontology. 2022;23:151–67. 10.1007/s10522-022-09949-y 35254570

[50] Lee JH, Jang JY, Kwon YH, Lee SH, Park C, Choi YH, et al. Effects of Rosemary extract on C2C12 myoblast differentiation and 5-aminoimidazole-4-cardoxamide ribonucleoside (AICAR)-induced muscle cell atrophy. Appl Sci. 2023;13:986.

[51] Chen KL, Li HX, Xu XL. The protective effect of rosmarinic acid on hyperthermia induced C2C12 muscle cells damage. Mol Biol Rep. 2014;41:5525–31. 10.1007/s11033-014-3429-6 24874305

[52] Sodagam L, Lewinska A, Kwasniewicz E, Kokhanovska S, Wnuk M, Siems K, et al. Phytochemicals rosmarinic acid, and ampelopsin, and amorfrutin-A modulate age-related phenotype of serially passaged human skin fibroblasts in vitro. Front Genet. 2019;10:81. 10.3389/fgene.2019.00081 PMC6394134 30847003

[53] Calabrese EJ, Dhawan G, Kapoor R, Agathokleous E, Calabrese V. Hormesis: Wound healing and fibroblasts. Pharmacol Res. 2022;184:106449. 10.1016/j.phrs.2022.106449 36113746

[54] Calabrese EJ, Dhawan G, Kapoor R, Agathokleous E, Calabrese V. Hormesis: Wound healing and keratinocytes. Pharmacol Res. 2022;183:106393. 10.1016/j.phrs.2022.106393 35961478

[55] Psotova J, Svobodova A, Kolarova H, Walterova D. Photoprotective properties of Prunella vulgaris and rosmarinic acid on human keratinocytes. J Photochem Photobiol B: Biology. 2006;84:167–74. 10.1016/j.jphotobiol.2006.02.012 16631374

[56] Gupta D, Archoo S, Naikoo SH, Abdullah ST. Rosmarinic acid: A naturally occurring plant-based agent prevents impaired mitochondrial dynamics and apoptosis in ultraviolet-B-irradiated human skin cells. Photochem Photobiol. 2022;98:925–34. 10.1111/php.13533 34608633

[57] Psotova J, Chlopeikova S, Miketova P, Simanek V. Cytoprotectivity of Prunella vulgaris on doxorubicin treated rat cardiomyocytes. Fitoterapia. 2005;76:556–61. 10.1016/j.fitote.2005.04.019 15972250

[58] Rahbardar MG, Eisvand F, Rameshrad M, Razavi BM, Hosseinzadeh H. In vivo and in vitro protective effects of rosmarinic acid against doxorubicin induced cardio toxicity. Nutr Cancer. 2022;74:747–60. 10.1080/01635581.2021.1931362 34085575

[59] Quan W, Liu H, Zhang W, Lou W, Gong Y, Yuan C, et al. Cardioprotective effect of rosmarinic acid against myocardial ischemia/reperfusion injury via suppression of the NF-ĸB inflammatory signalling pathway and ROS production in mice. Pharm Biol. 2021;59:220–9. 10.1080/13880209.2021.1878236 PMC7894452 33600735

[60] Yeni D, Inanc ME, Avdatek F, Tuncer PB, Cil B, Turkmen R, et al. Supplementation of rosemarinic acid has reduced oxidative stress on bull spermatozoa following the freeze thawing process. Cryo Letters. 2018;39:156–65. 29734425

[61] Motlagh MK, Sharafi M, Zhandi M, Mohammadi-Sangcheshmeh A, Shakeri M, Soleimani M, et al. Antioxidant effect of rosemary (Rosmarinus officialis L.) extract in soybean lecithin-based semen extender following freeze thawing process of ram sperm. Cryobiology. 2014;69:217–22. 10.1016/j.cryobiol.2014.07.007 25050864

[62] Feng TY, Lv DL, Zhang X, Du Y-Q, Yuan Y-T, Chen M-J, et al. Rosmarinic acid improves boar sperm quality, antioxidant capacity and energy metabolism at 17oC via AMPK activation. Reprod Domest Anim. 2020;55:1714–24. 10.1111/rda.13828 32969084

[63] Polat H, Kurtoglu IZ. Effect of antioxidants on cryopreserved turbot (Scophthalmus maximus) spermatozoa quality and DNA damage. Turkish J Fish Aquat Sci. 2023;23:TRJFAS22300.

[64] Zidni I, Lee HB, Yoon JH, Park JY, Oh YJ, Oh YD, et al. Effect of antioxidants in cryopreservation media on spotted halibut (Verasfer variegatus) sperm quality during cryopreservation. Aquaculture. 2022;557:738351.

[65] Lv M, Chen W, Weng S, Chen H, Cheng Y, Luo T. Rosmarinic acid compromises human sperm functions by an intracellular CA-2 plus concentration related mechanism. Reprod Toxicol. 2018;81:58–63. 10.1016/j.reprotox.2018.07.079 30009954

[66] Fialova SB, Kello M, Coma M, Slobodnikova L, Drobna E, Holkova I, et al. Derivatization of rosmarinic acid enhances its in vitro antitumor, antimicrobial and antiprotozoal properties. Molecules. 2019;24:1078. 10.3390/molecules24061078 PMC6470549 30893808

[67] Li H, Zhang Y, Chen H-H, Huang E, Zhuang H, Li D, et al. Rosmarinic acid inhibits stem-like breast cancer through hedgehog and Bcl-1/Bax signaling pathways. Pharmacog Mag. 2019;65:600–6.

[68] Yesil-Celikta O, Sevimli C, Bedir E, Vardar-Sukan F. Inhibitory effects of rosemary extracts, carnosic acid and rosmarinic acid on the growth of various human cancer cell lines. Plant Foods Hum Nutr. 2010;65:158–63. 10.1007/s11130-010-0166-4 20449663

[69] Sari U, Zaman F. Effects of rosmarinic acid and doxorubicine on an ovarian adenocarcinoma cell line (OVCAR3) via the EGFR pathway. Acta Cir Bras. 2024;39:e390524. 10.1590/acb390524 PMC10852540 38324801

[70] Ramanauskiene K, Raudonis R, Majiene D. Rosmarinic acid and Melissa officinalis extracts differently affect glioblastoma cells oxidative medicine and cellular longevity. Oxid Med Cell Longev. 2016;16:1564257. 10.1155/2016/1564257 PMC5027300 27688825

[71] Calabrese EJ. Cancer biology and hormesis: Human tumor cell lines commonly display hormetic (biphasic) dose responses. Crit Rev Toxicol. 2005;35:463–582. 10.1080/10408440591034502 16422392

[72] Calabrese EJ, Osakabe N, Di Paola R, Siracusa R, Fusco R, D'Amico R, et al. Hormesis defines the limits of lifespan. Aging Res Rev. 2023;91:102074. 10.1016/j.arr.2023.102074. 37709054

[73] Calabrese EJ, Nascarella M, Pressman P, Hayes AW, Dhawan G, Kapoor R, et al. Hormesis determines lifespan. Ageing Res Rev. 2024;94:102181. 10.1016/j.arr.2023.102181. 38182079

[74] Liman R, Cigerci IH, Gokce S. Cytogenetic and genotoxic effects of rosmarinic acid on Allium cepa L. root meristem cells. Food Chem Toxicol. 2018;121:444–9. 10.1016/j.fct.2018.09.022 30248483

[75] Zhang J-J, Wang Y-L, Feng X-B, Song X-D, Liu W-B. Rosmarinic acid inhibits proliferation and induces apoptosis of hepatic stellate cells. Biol Pharm Bull. 2011;34:343–8. 10.1248/bpb.34.343 21372382

[76] El-Lakkany NM, El-Maadawy WH, Seif el-Din SH, Hammam OA, Mohamed SH, Ezzat SM, et al. Rosmarinic acid attenuates hepatic fibrogenesis via suppression of hepatic stellate cell activation proliferation and induction of apoptosis. Asian Pac J Trop Med. 2017;10:444–53. 10.1016/j.apjtm.2017.05.012 28647181

[77] Shang R, Chen L, Xin Y, Wang G, Li S, Li L. Evaluation of rosmarinic acid on broiler growth performance, serum biochemistry, liver antioxidant activity, and muscle tissue composition. Animals. 2022;12:3313. 10.3390/ani12233313 PMC9739218 36496834

[78] Zhang Y, Guo J, Nie XW, Li ZY, Wang YM, Liang S, et al. Rosmarinic acid treatment during porcine oocyte maturation attenuates oxidative stress and improves subsequent embryo development in vitro. Peer J. 2019;7:6930. 10.7717/peerj.6930 PMC6587974 31249731

[79] Nieman KM, Sanoshy KD, Bresciani L, Schild AL, Kelley KM, Lawless AL, et al. Tolerance, bioavailability, and potential cognitive health implications of a distinct aqueous spearmint extract. Func Foods Health Dis. 2015;5:165–87.

[80] Hitl M, Kladar N, Gavaric N, Bozin B. Rosmarinic acid human pharmacokinetics and health benefits. Planta Med. 2021;87:273–82. 10.1055/a-1301-8648 33285594

[81] Nakazawa T, Ohsawa K. Metabolism of rosmarinic acid in rats. J Nat Prod. 1998;61:993–6. 10.1021/np980072s 9722482

[82] Baba S, Osakabe N, Natsume M, Terao J. Orally administered rosmarinic acid is present as the conjugated and or methylated forms in plasma and is degraded and metabolized to conjugated forms of caffeic acid, for every look acid and m-coumaric acid. Life Sci. 2004;75:165–78. 10.1016/j.lfs.2003.11.028 15120569

[83] Li X, Yu C, Lu Y, Gu Y, Lu J, Xu W, et al. Pharmacokinetics, tissue distribution, metabolism, and excretion of depside salts from Salvia miltiorrhiza and rats. Drug Metab Dispos. 2007;35:234–9. 10.1124/dmd.106.013045 17132761

[84] Chen JF, Bao X, Lin C, Zhou G. Pharmacokinetics of rosmarinic acid in rats and tissue distribution in mice. Am J Pharm. 2019;38:985–90.

[85] Baba S, Osakabe N, Natsume M, Yasuda A, Muto Y, Hiyoshi K, et al. Absorption, metabolism, degradation and urinary excretion of rosmarinic acid after intake of Perilla frutescens extract in humans. Eur J Nutr. 2005;44:1–9. 10.1007/s00394-004-0482-2 15309457

[86] Scholey A, Gibbs A, Neal C, Perry N, Ossoukhova A, Bilog V, et al. Anti-stress effects of lemon balm containing foods. Nutrients. 2014;6:4805–21. 10.3390/nu6114805 PMC4245564 25360512

[87] Hitl M, Pavlovi N, Brkic S, Dragovic G, Srdenovic-Conic B, Kladar N. Plasma concentrations of rosmarinic acid in patients on antiviral therapy: and silico exploration based on clinical data. Intern J Mol Sci. 2024;25:2230. 10.3390/ijms25042230 PMC10888967 38396908

[88] Dominguez-Avila JA, Wall-Medrano A, Velderrain-Rodriguiz GR, Chen CO, Salazar-Lopes NJ, Robles-Sanchez M, et al. Gastrointestinal interactions, absorption, splanchnic metabolism and pharmacokinetics of orally ingested phenolic compounds. Food Func. 2017;8:15–38. 10.1039/c6fo01475e 28074953

[89] Konishi Y, Hitomi Y, Yoshida M, Yoshioka Y. Pharmacokinetic study of caffeic and rosmarinic acids in rats after oral administration. J Agric Food Chem. 2005;53:4740–6. 10.1021/jf0478307 15941309

[90] Goodwin BL, Rutheven CR, Sandler M. Gut flora and the origin of some urinary aromatic phenolic compounds. Biochem Pharm. 1994;47:2294–7. 10.1016/0006-2952(94)90268-2 8031324

[91] Noguchi-Shinohara M, Ono K, Hamagichi T, Iwasa K, Nagai T, Kobayashi S, et al. Pharmacokinetics, safety and tolerability of Melissa officialis extract which contains rosmarinic acid and healthy individuals: A randomized controlled trial. Plos One. 2015;10:126422. 10.1371/journal.pone.0126422 PMC4433273 25978046

[92] Ritschel WA, Startzacher A, Sabouni A, Hussain AS, Koch HP. Percutaneous absorption of rosmarinic acid in the rat. Meth. Find. Exper Clin Pharm. 1989;11:345–52. 2755281

[93] Hase T, Shishido S, Yamamoto S, Yamashita R, Nukima H, Taira S, et al. Rosmarinic acid suppresses Alzheimer's disease development by reducing amyloid β aggregation by increasing monoamine secretion. Sci Rep. 2019;9:8711. 10.1038/s41598-019-45168-1 PMC6581955 31213631

[94] Ozevren H, Deveci E, Tuncer MC. The effect of rosmarinic acid on deformities occurring in brain tissue by craniectomy method. Histopathological evaluation of IBA one and GFP expressions. Octa Cr Bras. 2020;35:e202000406. 10.1590/s0102-865020200040000006 PMC7307720 32578724

[95] Kroon PA, Clifford MN, Crozier A, Day AJ, Donovan JL, Manach C, et al. How should we assess the effects of exposure to dietary polyphenols in vitro. Amer J Clin Nutr. 2004;80:15–21. 10.1093/ajcn/80.1.15 15213022

[96] Gonzales GB. In vitro bioavailability and cellular bioactivity studies of flavonoids and flavonoid rich plant extracts: questions, considerations and future perspectives. Proc Nutr Soc. 2017;76:175–81. 10.1017/S0029665116002858 27903318

[97] Scuto S, Fampulla F, Reali GM, Spano SM, Salinaro AT, Calabrese V. Hormetic nutrition and redox regulation in gut brain axis disorders. Antioxidants. 2024;13:484. 10.3390/antiox13040484 PMC11047582 38671931

